# The ATGL lipase cooperates with ABHD5 to mobilize lipids for hepatitis C virus assembly

**DOI:** 10.1371/journal.ppat.1008554

**Published:** 2020-06-15

**Authors:** Gabrielle Vieyres, Isabelle Reichert, Arnaud Carpentier, Florian W. R. Vondran, Thomas Pietschmann

**Affiliations:** 1 Institute of Experimental Virology, TWINCORE, Centre for Experimental and Clinical Infection Research; a joint venture between the Medical School Hannover (MHH) and the Helmholtz Centre for Infection Research (HZI), Hannover, Germany; 2 German Centre for Infection Research (DZIF), partner site Hannover-Braunschweig, Germany; 3 ReMediES, Department of General, Visceral and Transplant Surgery, Hannover Medical School, Hannover, Germany; University of California, San Diego, UNITED STATES

## Abstract

Lipid droplets are essential cellular organelles for storage of fatty acids and triglycerides. The hepatitis C virus (HCV) translocates several of its proteins onto their surface and uses them for production of infectious progeny. We recently reported that the lipid droplet-associated α/β hydrolase domain-containing protein 5 (ABHD5/CGI-58) participates in HCV assembly by mobilizing lipid droplet-associated lipids. However, ABHD5 itself has no lipase activity and it remained unclear how ABHD5 mediates lipolysis critical for HCV assembly. Here, we identify adipose triglyceride lipase (ATGL) as ABHD5 effector and new host factor involved in the hepatic lipid droplet degradation as well as in HCV and lipoprotein morphogenesis. Modulation of ATGL protein expression and lipase activity controlled lipid droplet lipolysis and virus production. ABHD4 is a paralog of ABHD5 unable to activate ATGL or support HCV assembly and lipid droplet lipolysis. Grafting ABHD5 residues critical for activation of ATGL onto ABHD4 restored the interaction between lipase and co-lipase and bestowed the pro-viral and lipolytic functions onto the engineered protein. Congruently, mutation of the predicted ABHD5 protein interface to ATGL ablated ABHD5 functions in lipid droplet lipolysis and HCV assembly. Interestingly, minor alleles of ABHD5 and ATGL associated with neutral lipid storage diseases in human, are also impaired in lipid droplet lipolysis and their pro-viral functions. Collectively, these results show that ABHD5 cooperates with ATGL to mobilize triglycerides for HCV infectious virus production. Moreover, viral manipulation of lipid droplet homeostasis via the ABHD5-ATGL axis, akin to natural genetic variation in these proteins, emerges as a possible mechanism by which chronic HCV infection causes liver steatosis.

## Author summary

The multifaceted role of lipid droplets and their position as the main metabolic hub in the cell makes them a coveted target for intracellular pathogens, be there parasites, bacteria or viruses. Lipid droplets are the main energy reservoir of the cell, and this is often the function hijacked by the pathogens. With its low density, its apolipoprotein coat and its neutral lipid core, the HCV particle resembles host-circulating lipoproteins. Here we report that HCV hijacks the lipid droplet-associated adipose triglyceride lipase (ATGL) for its morphogenesis. Given that ABHD5 and to some extent ATGL also participate in lipoprotein secretion, our results indicate that lipid droplets might serve as a source for the lipids of the atypical HCV lipo-viro-particle. They also imply that viral hijacking of the ABHD5-ATGL host machinery might disturb lipid droplet homeostasis thus contributing to HCV-dependent liver steatosis.

## Introduction

Lipid droplets are the main intracellular neutral lipid storage reservoir and energy source [1]. They are also dynamic organelles and capable of regulating the mobilization of the lipid stores depending on lipid availability and energy needs in the organism. Excessive or deficient neutral lipid storage can cause a spectrum of human diseases, with genetic or multifactorial etiologies [2]. In particular, one fourth of the global population has non-alcoholic fatty liver disease (NAFLD) and, therefore, is pre-disposed to develop liver cirrhosis or hepatocellular carcinoma. This prevalence is still rising, mostly due to the obesity epidemic [3]. Hepatitis C virus infection is a further risk factor for liver steatosis, with about half the chronically infected patients affected [4]. Despite the commercialization of efficient antivirals, hepatitis C virus (HCV) still chronically infects 71 million people worldwide [5]. Together with hepatitis B virus, it constitutes a public health threat that the World Health Organization plans to eliminate by 2030 [6]. A vaccine might be necessary to reach this ambitious goal [7] but vaccine development faces major hurdles, because HCV has evolved multiple ways to evade neutralizing antibodies. For instance, the hypervariable regions [8] and numerous glycosylation sites–the so called glycan coat–mask conserved epitopes of the viral envelope proteins E1 and E2 involved in receptor interactions [9]. Moreover, HCV particles incorporate serum lipoproteins, which enhances virus cell surface binding, and the kinetics of cell entry thereby facilitating antibody escape [10]. Given the tight interaction of HCV with lipoproteins, HCV viruses have been designated as “lipo-viro-particles” [11]. However, several aspects of how these lipo-viro-particles are built in the infected cells remain unclear.

HCV is a plus-strand RNA virus belonging to the *Flaviviridae* family. Its single polyprotein is associated upon synthesis with the endoplasmic reticulum (ER) membrane, where the envelope glycoproteins E1 and E2 remain resident [12]. Components of the viral replication complex reorganize ER membranes into a replication organelle, which is composed mostly of double-membrane vesicles and called the membranous web [13, 14]. Additionally, the HCV capsid protein core, synthesized at the ER membrane, is translocated after cleavage by the signal peptidase and the signal peptide peptidase to the lipid droplet surface [15, 16], thereby initiating viral morphogenesis [17]. Thus, HCV assembly takes place at a virus-induced interface between (i) the membranous web which protects the replication complexes and generates newly synthesized RNAs, (ii) the ER membrane where viral glycoproteins are embedded and (iii) the lipid droplets that are coated with core and NS5A [18]. Importantly, apolipoprotein E (ApoE) is an essential co-factor for HCV assembly [19, 20] and is recruited both intracellularly and after virus secretion to the virion [21–23]. This suggested that HCV might take the lipoprotein secretion route to exit the cell (reviewed in [24]). The role of ApoE in virion morphogenesis can be substituted by other exchangeable apolipoproteins [25, 26] but also by ApoB [26], by the pestiviral E^rns^ or the flaviviral NS1 protein [27] or by a range of small secretory proteins [28]. Most of these proteins are secreted in association with a neutral lipid core [24], suggesting that HCV morphogenesis and secretion itself might depend on the recruitment of neutral lipids, which are indeed largely overrepresented in the lipo-viro-particle [24, 29].

We previously characterized ABHD5 as a host factor involved in HCV assembly and release [30]. ABHD5 associates with lipid droplets [31] and adopts an α/β hydrolase fold like many serine hydrolases [32]. With an asparagine residue replacing the usual catalytic serine, ABHD5 however has no intrinsic lipase activity [33] but it can activate lipases, in particular adipose triglyceride lipase (ATGL, also known as patatin-like phospholipase domain-containing protein 2, PNPLA2) in adipocytes [34]. Therefore, ABHD5 is designated as a co-lipase or lipase co-factor. Here we further unravelled the mechanisms by which ABHD5 functions in HCV assembly. We identified ATGL as ABHD5´s main effector in hepatocyte-derived cells and a new host factor involved in HCV production. Furthermore, as for ABHD5 [30], minor alleles of ATGL associated with human lipid storage diseases [35] do not support HCV production, suggesting a broader overlap between host determinants of liver steatosis and of HCV morphogenesis.

## Results

### ATGL is the most likely ABHD5 partner in HCV morphogenesis and hepatic lipid droplet consumption

**Fig 1:**
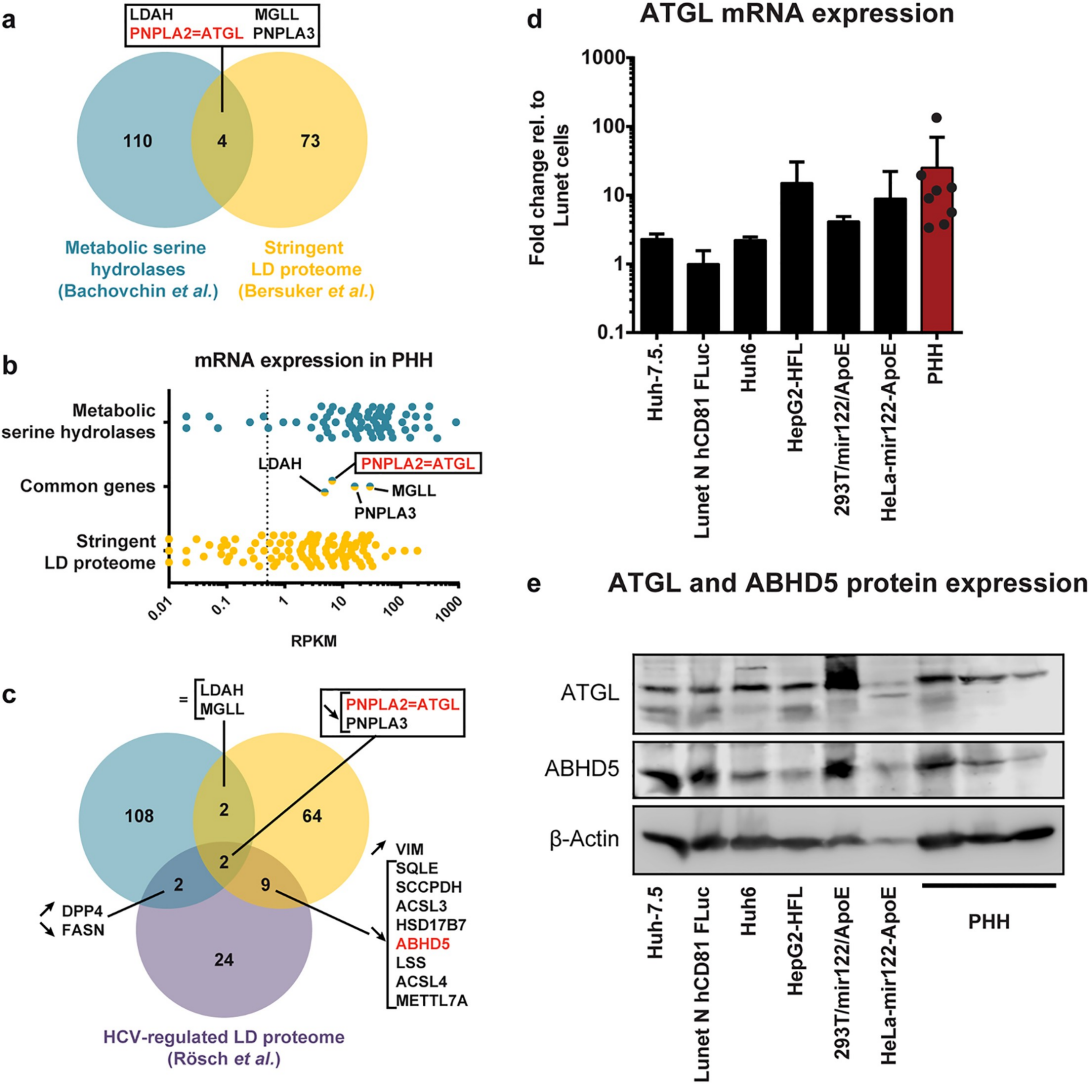
Unbiased search for lipid droplet-associated lipases involved in HCV assembly. (**a**) Venn diagram of metabolic serine hydrolases [32] and the Huh-7 lipid droplet proteome [38]. (**b**) mRNA expression of these genes was determined in primary human hepatocytes (PHHs) from 3 donors by RNA-seq (NCBI database, GEO accession number GSE132548, (Tegtmeyer B, Vieyres G, submitted for publication)). The vertical dotted line indicates an arbitrary expression threshold of 0.5 RPKM (reads per kilobase per million mapped reads). (**c**) Venn diagram depicting the fraction of proteins from (a), whose lipid droplet association varies upon HCV infection according to Rösch *et al*. [39] (purple circle). Arrows pointing upwards, arrows pointing downwards and the equal sign indicate respectively an increased, decreased or unchanged lipid droplet association upon HCV infection [39]. (**d**) ATGL mRNA expression in a panel of cell lines and in PHHs from 8 individual donors, as determined by qRT-PCR. (**e**) ABHD5 and ATGL protein expression in the same cell lines and in PHHs from 3 random donors.

In our previous study, all mutations impairing ABHD5 co-lipase activity also hindered its pro-viral effect [30]. In particular, mutation of the tribasic lipid droplet consumption motif (TBLC), KRK233-235, specifically abrogated ABHD5 pro-lipolytic and pro-viral functions, without affecting the protein association with the lipid droplets [30]. This correlation between pro-lipolytic and pro-viral functions of ABHD5 suggested that ABHD5 participates in HCV morphogenesis by recruiting the triglycerides from the lipid droplets and that it exerts its role via the activation of a lipase. The aim of this study was the identification of the lipase cooperating with ABHD5 in HCV production. The ATGL lipase is a known effector of ABHD5 [34] and its enzymatic activity is multiplied up to 20-fold by the co-lipase *in vitro*. In cells however, ATGL was mostly studied in adipocytes [34, 36]. In the liver, ATGL is expressed at low levels and its physiological role remains unclear [37] (see Discussion). We therefore used an unbiased approach searching the entire family of serine hydrolases encompassing around 240 proteins, as reviewed by Bachovchin *et al*. [32] for candidate ABHD5 effectors. Half the family members are serine proteases, so we focused on the other half, consisting of 114 human metabolic serine hydrolases [32]. Next, we conducted a gene ontology (GO) analysis of these 114 metabolic serine hydrolases and confirmed that these included 38 lipases (S1 Fig). Most of them are organized around an α/β hydrolase fold with a Serine-Histidine-Aspartic acid catalytic triad, but other types of lipases exist, for instance those with a patatin domain and a Serine-Aspartic acid diad (e.g. ATGL) [32]. Out of these 114 metabolic serine hydrolases, a proteomic study had shown 4 of these proteins to be enriched at the lipid droplet surface in Huh-7 cells [38] (Fig 1A). These proteins include lipid droplet associated hydrolase (LDAH), monoglyceride lipase (MGLL), and patatin like phospholipase domain containing 2 and 3, also known as ATGL and ADPN, respectively. All 4 proteins were readily expressed at the mRNA level in primary human hepatocytes (Fig 1B). Interestingly, Rösch and colleagues reported that HCV infection displaces both PNPLA2 (another name for ATGL) and PNPLA3 (also known as adiponutrin [ADPN]) from the surface of lipid droplets, similarly to the ABHD5 co-lipase (Fig 1C) [39]. Furthermore, in a functional STRING network analysis, ATGL was the only protein directly linked to the ABHD5 co-lipase (S1B Fig). We could also confirm ATGL expression at the mRNA (Fig 1D) and protein levels (Fig 1E) in a panel of cell lines permissive for HCV production as well as in primary human hepatocytes (PHHs) of multiple donors. For these reasons, we decided to analyse in detail the contribution of ATGL to HCV assembly and hepatic lipid droplet hydrolysis and to unravel its interplay with ABHD5.

### ATGL associates with lipid droplets together with ABHD5 and G0S2

**Fig 2:**
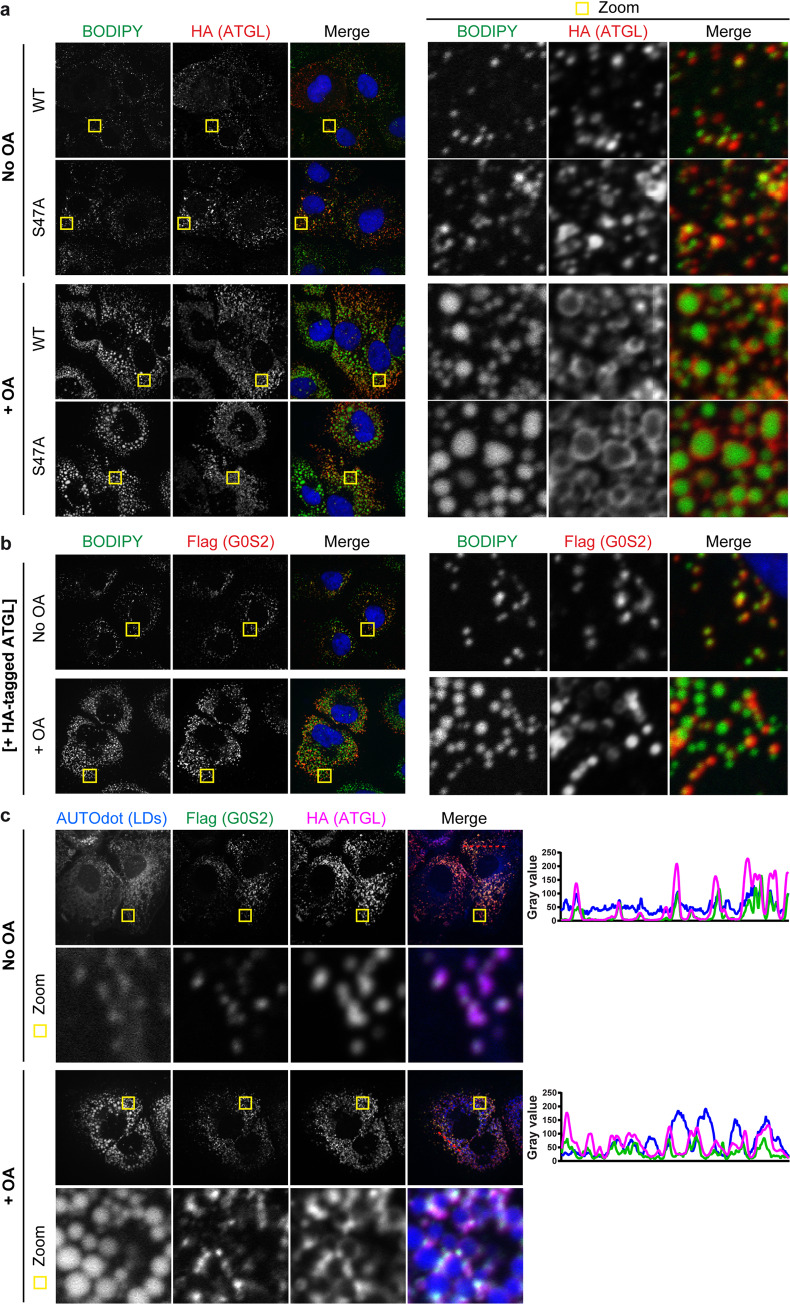
ATGL and G0S2 colocalize at the lipid droplet surface. ATGL and G0S2 were expressed by lentiviral transduction and detected by indirect immunofluorescence using antibodies against the respective epitope tag. Colocalization between the proteins or with the lipid droplet markers (BODIPY 493/503 or AUTOdot [42]) was tested in Lunet N hCD81 cells cultured with or without oleic acid (OA), as indicated. (**a**) Subcellular localization of HA-tagged ATGL WT or S47A relative to lipid droplets. (**b**) Subcellular localization of Flag-tagged G0S2 relative to lipid droplets. Note that we had to co-transduce ATGL (here HA-tagged ATGL, which was left unstained) to achieve a robust and reproducible G0S2 detection. (**c**) Subcellular localization of Flag-tagged G0S2 relative to HA-tagged ATGL and lipid droplets. The plots on the right side indicate the intensity profiles in the different channels along the red dotted line depicted in the merge picture.

**Fig 3:**
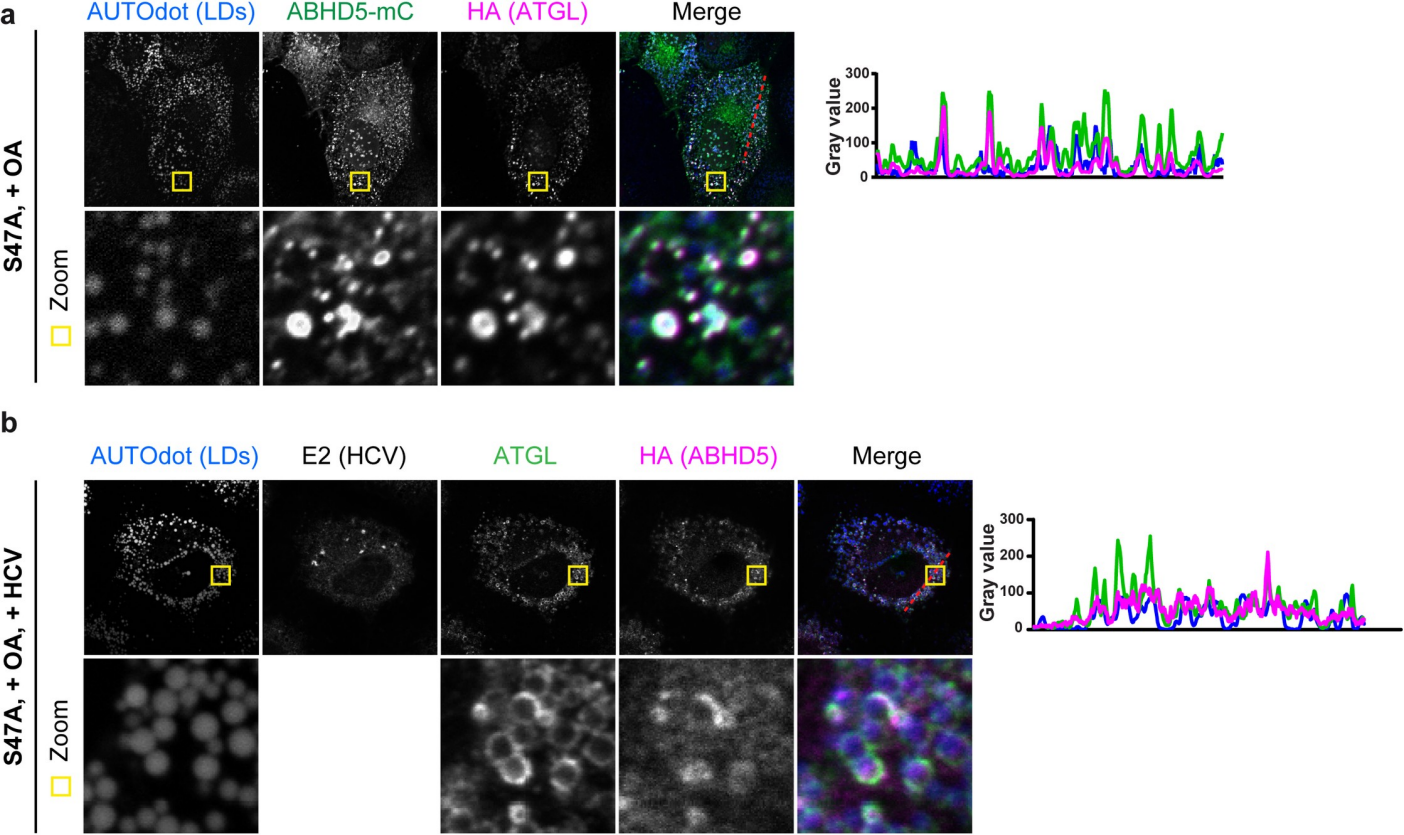
ABHD5 and ATGL colocalize at the lipid droplet surface. ABHD5 and ATGL S47A were expressed by lentiviral transduction and detected by indirect immunofluorescence using antibodies against the respective epitope tag or by their auto-fluorescence in case of the ABHD5-mCitrine fusion protein. Colocalization between the proteins or with the lipid droplet marker AUTOdot was tested in naive (**a**) or HCV-infected (**b**) Lunet N hCD81 cells after oleic acid (OA) induction. (**a**) Subcellular localization of HA-tagged ATGL S47A relative to ABHD5-mCitrine (mC) and lipid droplets. (**b**) Subcellular localization of HA-tagged ABHD5 relative to ATGL S47A and lipid droplets, in HCV-infected cells. HCV infection was verified by immunostaining against HCV E2 glycoprotein. In (**a**) and (**b**) the plots on the right side indicate the intensity profiles in the different channels along the red dotted line depicted in the merge picture.

Having confirmed ATGL expression in cell lines and primary cells relevant for HCV infection (Fig 1D and 1E), we investigated its subcellular localization relative to the lipid droplets and the ABHD5 co-lipase. Commercially available antibodies could not robustly visualize endogenous expression of ATGL, so we tagged ATGL with a double HA-epitope and examined protein localization upon lentiviral transduction. HA-tagged ATGL was mostly cytoplasmic and colocalized with lipid droplets (Fig 2A, 1^st^ row), forming the typical ring pattern when lipid droplets were induced by oleic acid treatment (Fig 2A, 3^rd^ row). This pattern was preserved for the catalytic serine mutant ATGL S47A, which lacks lipase activity [34, 40] (Fig 2A, rows 2 and 4). G0S2 is an endogenous inhibitory peptide of ATGL [41]. When we over-expressed Flag-tagged G0S2 by lentiviral gene transfer, detection was weak and not reproducible. However, when we co-expressed Flag-GOS2 with HA-tagged ATGL, G0S2 was detectable and co-localized with lipid droplets (Fig 2B). Furthermore, G0S2 co-localized with ATGL at the lipid droplet surface (Fig 2C). We then investigated ATGL colocalization with ABHD5. Upon co-expression of lipase and co-lipase, the cells had very few detectable lipid droplets and therefore ABHD5 and ATGL were mostly dispersed in the cytoplasm, although colocalization puncta were detected in cells that had lipid droplet remnants. We therefore did not include the data here but decided to study the colocalization using the catalytic site mutant of ATGL (S47A) and in oleic acid-treated cells. In these conditions, ABHD5-mCitrine robustly associated with the ATGL S47A mutant around lipid droplets (stained with the AUTOdot dye [42]) (Fig 3A) and this association was preserved in HCV-infected cells (Fig 3B). These results indicate that ATGL can localize with the ABHD5 co-lipase and with its G0S2 inhibitor at the lipid droplet surface in hepatoma cells, suggesting that the 3 proteins can cooperate in these cells.

### ATGL participates in hepatic lipid droplet lipolysis and in HCV assembly

**Fig 4:**
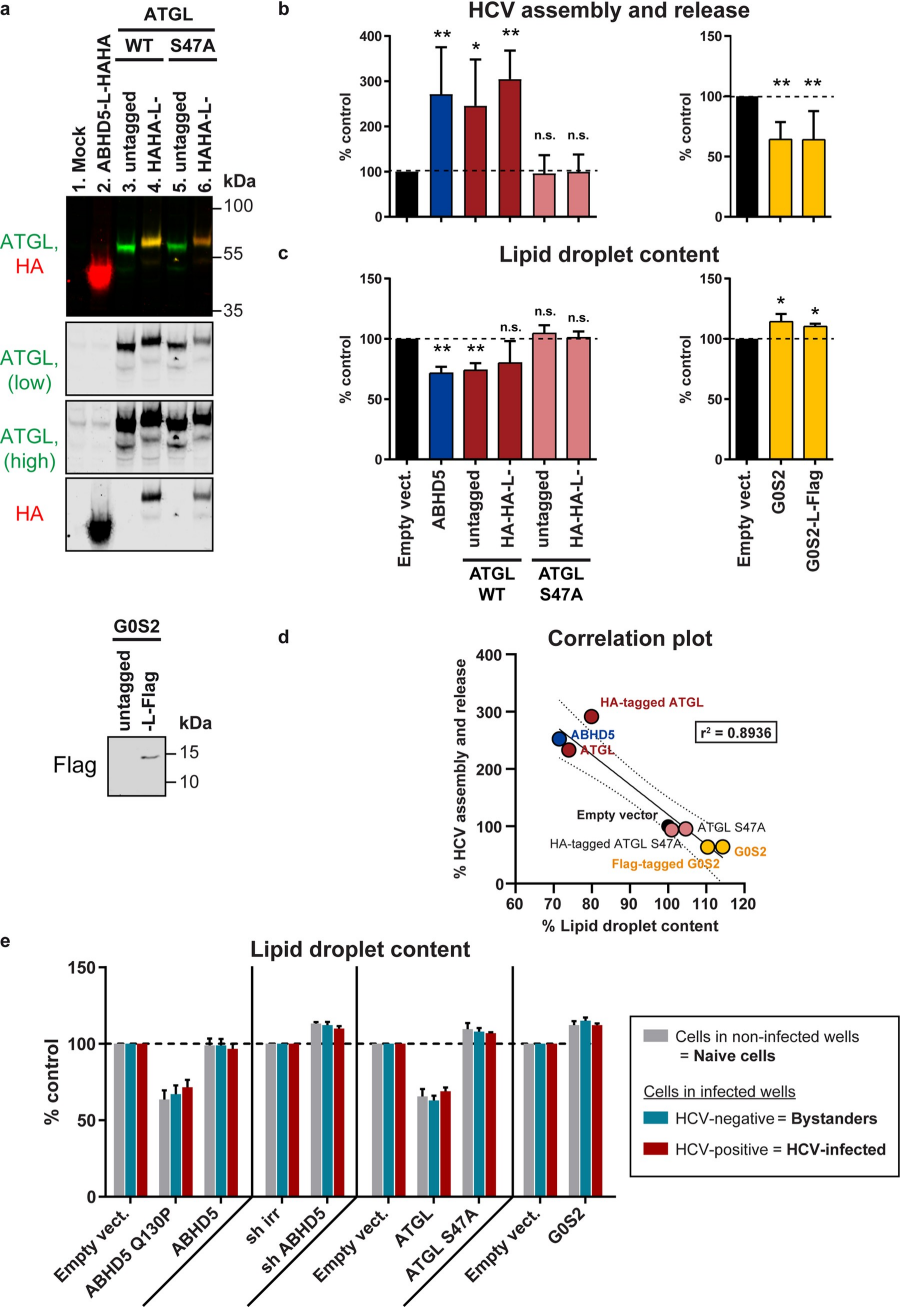
Endogenous or ectopically expressed ATGL controls lipid droplet lipolysis and HCV assembly. (**a**) Western blot verification of ABHD5, ATGL and G0S2 expression 72 hours after lentiviral transduction in Lunet N hCD81 cells. Proteins were detected with antibodies directed against ATGL, or the HA and Flag epitopes. For ATGL detection, the endogenous protein can be seen in the high contrast picture (lanes 1 and 2). (**b**) Effect of ABHD5, ATGL or G0S2 expression on HCV assembly and release. HCV production was determined in a whole replication cycle assay with the JcR2a virus in Lunet N hCD81 Fluc cells and normalized for replication (n = 6 for left panel, n = 7 for right panel). (**c**) Effect of ABHD5, ATGL or G0S2 expression on lipid droplet lipolysis. Lipid droplet content was measured by flow cytometry 72 hours post-lentiviral transduction in Lunet N hCD81 cells (n = 4 for all conditions except HA-tagged ATGL constructs and Flag-tagged G0S2 where n = 3). (**d**) Correlation between HCV production and lipid droplet lipolysis. This graph gathers the results plotted in panels b and c as well as the linear regression (full line), 95% confidence band (dotted lines) and r^2^ value. Data in panels b, c and d were normalized to empty vector- transduced cells. (**e**) Effect of ABHD5, ATGL or G0S2 expression on lipid droplet lipolysis in naive, bystanders and HCV-infected cells. Lunet N hCD81 cells were lentivirally transduced with the constructs indicated on the X axis and infected 48 hours later with HCV Jc1. Two days later, the cells were harvested, mixed with mRuby2-expressing reference cells and fixed, as explained in S2A Fig. In this case however, the cells were permeabilized and stained with an antibody against HCV NS5A to determine their infection status, in addition to the lipid droplet dye BODIPY. Naive, bystanders and HCV-infected cells were gated as depicted in S3B Fig. Their lipid droplet content was measured and normalized for the reference cell population, to correct for staining or measurement variations. For each condition, the results were then normalized to the respective empty vector-transduced cell population (n = 3).

**Fig 5:**
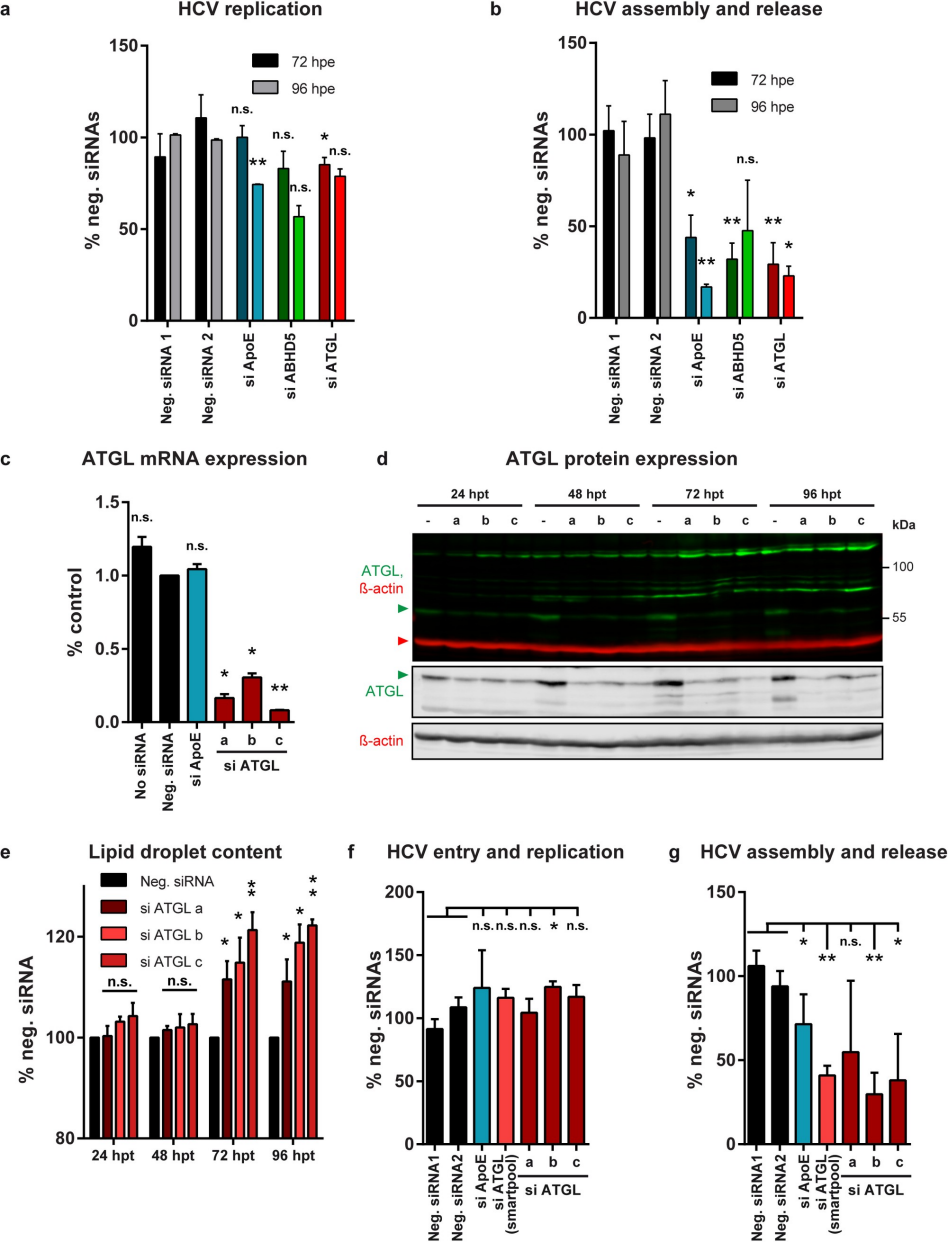
ATGL knockdown induces lipid droplet accumulation and reduces HCV assembly. (**a, b**) Effect of pools of 3 siRNAs targeting ApoE or ABHD5 or 4 siRNAs targeting ATGL (SMARTpool) on HCV replication cycle (n = 3 for 72 hours post-electroporation (h.p.e.) and n = 2 for 96 h.p.e.). Lunet N hCD81 FLuc cells were electroporated with HCV RNA and transfected 4 hours later with the siRNA pools. Panel a represents the effects on the early replication events (RLUs in the producer cells). Panel b depicts the effects on HCV production (RLUs in the target cells normalized by RLUs in the producer cells). For the statistics, we compared the results obtained with the different siRNAs to the average of the negative siRNAs 1 and 2. (**c-g**) Effect of single siRNAs targeting ATGL (si ATGL a, b and c) on ATGL expression, lipid droplet content and HCV replication cycle. (**c**) ATGL mRNA expression was assessed 96 hours (h) post-transfection by qRT-PCR and normalized to GAPDH (n = 2). (**d**) ATGL protein expression was verified by Western blot. The green and red arrowheads point at the ATGL and β-actin protein bands. (**e**) The lipid droplet content of cells transfected with ATGL-specific or control siRNAs was evaluated by flow cytometry at 24-48-72-96 hours post-siRNA transfection. (**f, g**) HCV JcR2a whole replication cycle was examined in the siRNA-transfected cells. (**f**) Entry and replication correspond to the RLuc reading in the producer cells at 48 h.p.i. normalized for the FLuc readings at the same time point, which reflect the cell viability and proliferation (n = 3, except SMARTpool where n = 2). (**g**) Assembly and release values are obtained by normalizing the RLuc readings in the target cells (infected with the 96 h.p.e. supernatants) by the RLuc readings in the producer cells at 48 h.p.e. (n = 4, except SMARTpool where n = 3).

To assess the role of ATGL in hepatic lipid droplet lipolysis and HCV production, we then over-expressed untagged and epitope-tagged ATGL, or repressed the functioning of the endogenous protein by over-expressing its G0S2 inhibitor (Fig 4). Ectopic expression of these proteins was confirmed by immuno-blotting (Fig 4A). In addition, we also silenced ATGL expression by RNA interference (Fig 5). The experimental setup for quantifying HCV assembly and lipid droplet lipolysis was described recently [30], and is summarized in S2 Fig. Briefly, we assessed lipolysis by staining cellular neutral lipids (mostly contained within the lipid droplets [1]) with the BODIPY dye and measuring the mean fluorescent BODIPY intensity per cell by flow cytometry. A reduction in BODIPY signal therefore corresponds to an increase in lipolysis, and vice versa. Ectopic over-expression of ATGL boosted HCV production more than 2-fold (Fig 4B and S3A–S3C Fig) and depleted the cellular lipid droplets by 20–24% (Fig 4C), mimicking the effect of ABHD5 over-expression. In contrast, ectopic over-expression of the catalytic dead mutant ATGL S47A had no effect on HCV assembly or lipid droplet lipolysis (Fig 4A and 4B). Moreover, G0S2 expression decreased lipid droplet lipolysis and HCV production (Fig 4B and 4C, right panels) emphasizing the role of endogenous ATGL in these cellular and viral functions. Importantly, HCV production correlated with lipid droplet lipolysis (decrease in the lipid droplet content) over the different conditions (Fig 4D). We next tested whether HCV infection modulated ATGL-dependent lipolysis (Fig 4E and S3D–S3F Fig). To this end, we modulated ATGL activity by targeting the lipase itself, the co-lipase ABHD5 or the lipase inhibitor G0S2. We then infected or not the cells with HCV and analysed the lipid droplet content of the cells 2 days later. At this time, in the infected wells, about half the cells scored positive for HCV NS5A immunostaining (S3D Fig), and we could compare the lipid droplet content of these HCV-positive cells to their neighbouring bystander cells and to the naive cells in non-infected wells (S3E Fig). In all tested conditions, HCV infection lead to an accumulation of lipid droplets (S3F Fig). Furthermore, the modulation of ATGL activity regulated lipid droplet lipolysis in a similar manner and extent in naive, infected and bystander cells (Fig 4E).

Next, we targeted ATGL expression by siRNA-mediated knockdown. We first used a pool of four siRNAs targeting ATGL. Progeny virus production was strongly impaired (81–85% reduction), comparable to cells treated with siRNAs targeting known HCV assembly co-factors (si ApoE, si ABHD5) (Fig 5B). Of note, ATGL and ApoE knockdown also affected HCV replication in these conditions, although to a lesser extent (15–21% reduction for si ATGL). This is likely because some virus had already spread at these timepoints. Three individual siRNAs targeting ATGL also potently decreased the mRNA and protein levels (Fig 5C and 5D) and lead to a time-dependent lipid droplet accumulation (Fig 5E). This time, early steps in HCV replication cycle were mostly unaffected or boosted (si ATGL b) (Fig 5F), likely because we measured an earlier timepoint (48 h.p.i.), but virus production decreased for all tested siRNAs (Fig 5G).

**Fig 6:**
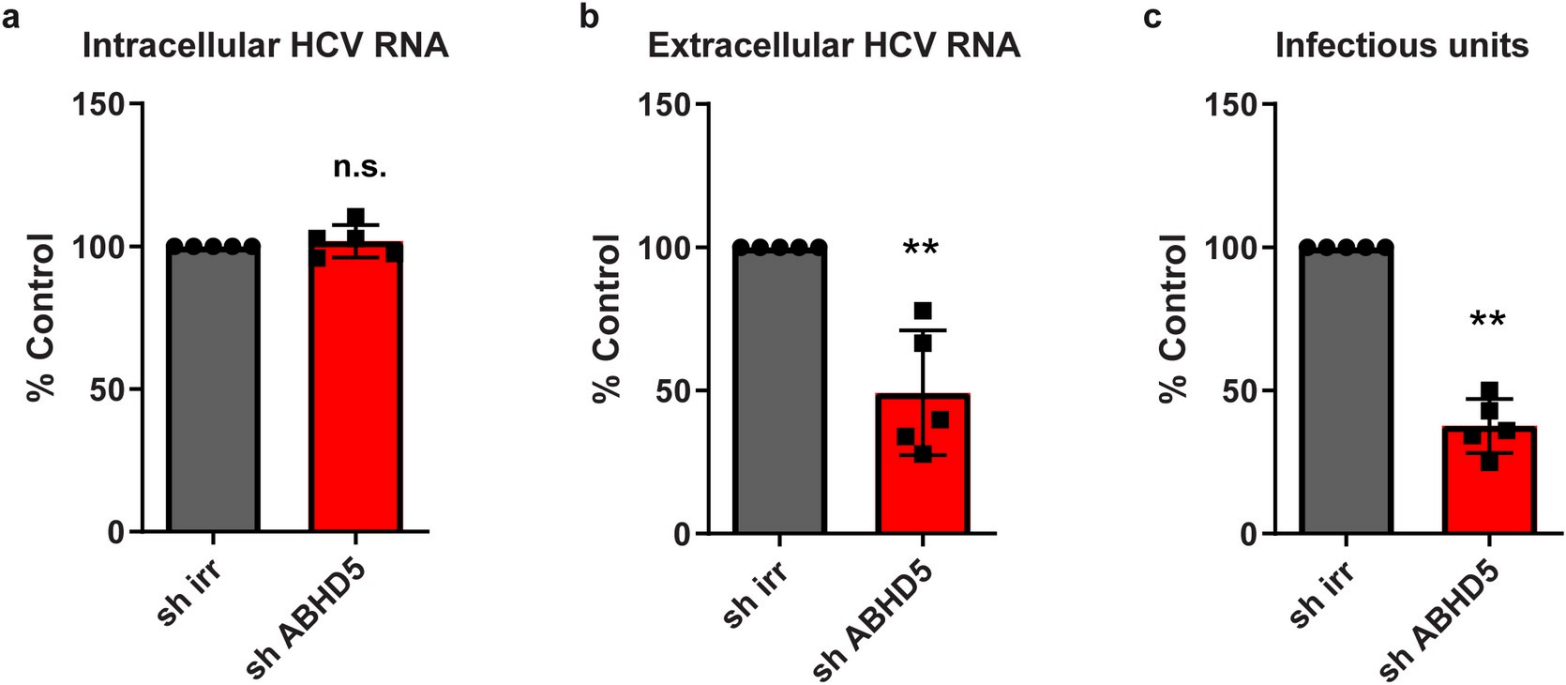
WT HCV also depends on ATGL-mediated lipid droplet. Lunet N hCD81 cells were lentivirally transduced with an ABHD5-targeting or irrelevant shRNA and infected 72 hours later with HCV Jc1. We harvested 48 hours later the cell lysates and supernatants (n = 5). (**a**) Intracellular HCV RNA was quantified from the cell lysates by qRT-PCR and normalized to the GAPDH housekeeping gene. (b) Extracellular HCV RNA was quantified from the cell culture supernatants by qRT-PCR. (c) Released infectivity was assessed by transferring the supernatants onto Huh-7.5 cells, culturing the cells for 48 hours and immunostaining them for HCV NS5A. We quantified the infected foci automatically as described in the Methods section.

As a further validation, we verified that wild-type HCV, without reporter gene, was also dependent on ATGL/ABHD5-dependent lipolysis. We knocked down ABHD5 expression and infected the cells with HCV Jc1. The accumulation of intracellular HCV RNA was not affected, suggesting that HCV entry and replication were not modulated (Fig 6A). However, depletion of the ABHD5 co-lipase reduced the amount of secreted HCV genome and infectivity (Fig 6B and 6C), suggesting that Jc1, like the reporter virus JcR2a, needs ATGL-driven lipolysis for virus assembly and / or release.

### Variants of ATGL associated with neutral lipid storage disease do not support HCV assembly nor lipid droplet lipolysis

**Fig 7:**
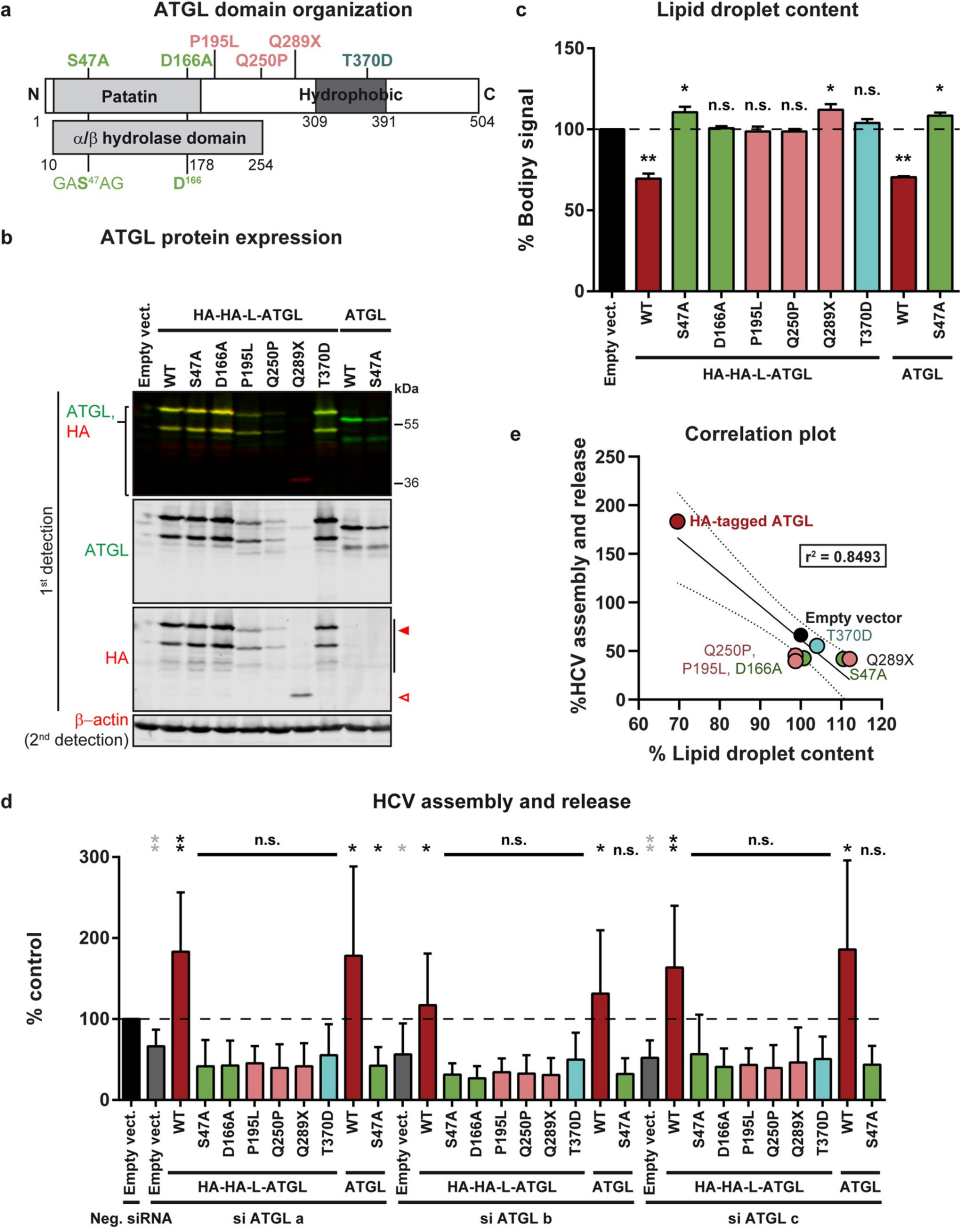
Pathogenic variants of ATGL do not support HCV assembly nor lipid droplet lipolysis. (**a**) Domain organization of human ATGL and description of the mutants used in this study. The patatin domain is conserved in a large family of hydrolases found in eukaryotes and bacteria [32] and is included in an α/β hydrolase domain. It comprises the two putative residues of the catalytic diad (S47 in the conserved GXSXG sequence and D166). Coding polymorphisms associated with NLSDM [35] and tested in this study are indicated in pink. Q289X corresponds to a premature stop codon. The T370D mutation is a phosphomimetics described to eliminate lipid droplet association of the mouse ATGL (T372D) without compromising the hydrolase activity [43]. (**b**) Western blot verification of the protein expression of the various ATGL variants in Lunet N hCD81 Fluc cells 7 days post-lentiviral transduction, at the time when HCV replication and production were measured. The full red arrowhead indicates the full-length HA-tagged ATGL whereas the empty red arrowhead points at the Q289X truncation mutant. Note that the upper band (around 56 kDa for the untagged ATGL) corresponds to the reported molecular weight of ATGL [44]. The second lower band could be a cleavage product of ATGL corresponding to the N-terminal part of the protein, since it is detected by the anti-HA antibody. We also observed this second band when staining the endogenous protein (see empty vector as well as Fig 4B, band just over the β-actin (42 kDa)). Although this band was to our knowledge not described from the literature, a fragment of around the same size could be seen in [45] (see Fig 1C for instance) as well as in the datasheets of several commercial anti-ATGL antibodies. (**c**) Cellular lipid droplet content in Lunet N hCD81 cells upon ectopic expression of untagged or HA-tagged ATGL variants, as measured by BODIPY 493/503 staining and flow cytometry (n = 3). (**d**) HCV assembly and release in Lunet N hCD81 Fluc cells upon ATGL knockdown with si ATGL a, b or c and rescue of ATGL expression with siRNA-resistant tagged or untagged ATGL variants. The horizontal dotted line corresponds to the control (neg. siRNA and empty vector). Statistics in grey highlight the knockdown effects and test differences between the different knockdowns (si ATGL a/b/c + empty vector, in grey) and the control (black bar). Statistics in black correspond to the rescue experiment and show differences to the respective grey bar (respective siRNA + empty vector) (n = 8). (**e**) Correlation between HCV production and lipid droplet lipolysis. This graph gathers the results plotted in panels c and d as well as the linear regression (full line), 95% confidence band (dotted lines) and r^2^ value. Note that the lipid droplet content is tested in an over-expression assay (X axis), whereas HCV assembly and release are assessed in the context of the rescue of an ATGL knockdown (Y axis). This is why the empty vector is set at 100% on the X axis, but at a lower value for the Y axis (presence of ATGL siRNA). (**c, d, e**) WT ATGL sequences (tagged or untagged) are indicated in dark red, the catalytic site mutants are in green, the clinical variants in pink and the phosphomimetics T370D mutant in light blue.

**Fig 8:**
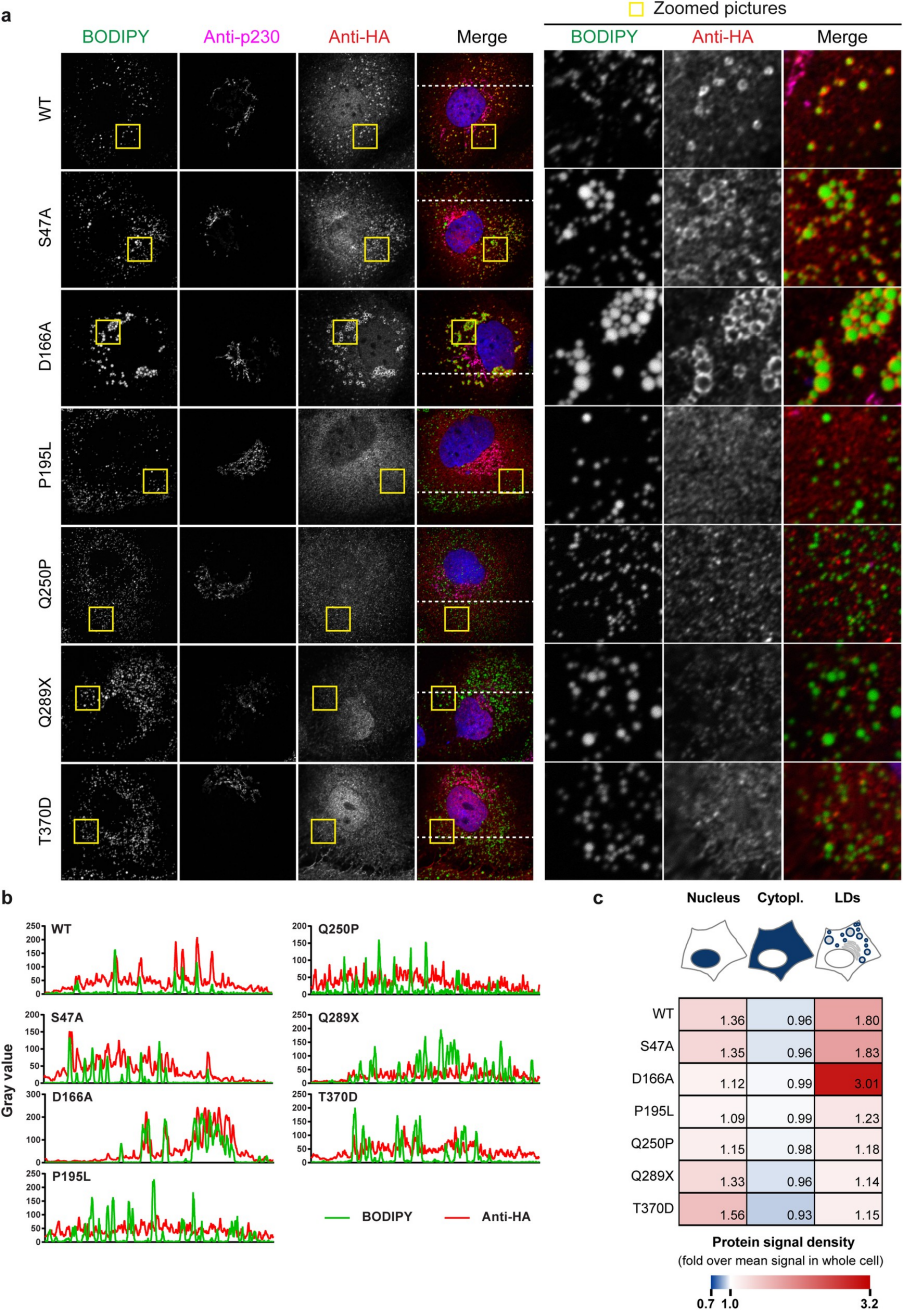
Subcellular localization and lipid droplet association of ATGL variants. (**a**) Immunofluorescence analysis of HA-tagged ATGL localization respectively to the lipid droplets (BODIPY 493/503) and *trans*-Golgi (p230 marker) in Lunet N hCD81 cells. (**b**) Intensity profiles of the green and red channels along the dotted line depicted in the merge pictures of panel a. (**c**) Quantification of ATGL accumulation in the nucleus, the cytoplasm and at the lipid droplet periphery. The heatmap shows the fold enrichment of the protein signal in each compartment as compared to the mean protein signal in the whole cell. Average of 3 experiments with at least 10 pictures per experiment.

In a next step, we examined the ATGL motifs necessary for its pro-viral and pro-lipolytic activities (Fig 7A). We disrupted ATGL enzymatic activity by mutating individually the serine and aspartic acid residues of the catalytic dyad. We also replaced the threonine residue in the predicted hydrophobic domain by an aspartic acid. This phosphomimetics prevents mouse ATGL from associating with lipid droplets but does not affect its enzymatic activity *in vitro* [43]. Finally, we tested two missense mutations (P195L and Q250P) and one nonsense mutation (Q289X, having a premature stop codon instead of Q289) associated with neutral lipid storage disease with myopathy (NLSDM) [35]. All mutants were readily expressed, although the pathogenic variants reached lower expression levels (Fig 7B). Wild-type ATGL efficiently degraded the lipid droplets and this function was not impaired by the double HA tag. However, none of the tested mutants was capable of hydrolysing the lipid droplets, and some even tended to increase the lipid droplet content (Fig 7C). In a virus complementation assay, wild-type ATGL rescued HCV production in ATGL-depleted cells (Fig 7D), without altering other steps of HCV replication cycle (S4 Fig). The ATGL mutants however did not rescue virus production (Fig 7D). The catalytic dead mutants (S47A and D166A) efficiently associated with lipid droplets (Fig 8A, rows 2 and 3), although D166A seemed to lead to lipid droplet clustering. However, the pathogenic variants and the T370D phosphomimetics were more diffuse in the cytoplasm, suggesting that both the catalytic activity and the lipid droplet association are crucial for ATGL’s functions (Fig 8 and S5 Fig). Interestingly, a fraction of ATGL was present in the cell nucleus, and this nuclear accumulation decreased upon oleic acid treatment (S5 Fig) but increased for the T370D mutant (Fig 8). This is reminiscent of the nuclear concentration of the Chanarin-Dorfman syndrome and engineered ABHD5 mutants that are incapable of binding the lipid droplets [30], and suggests that ABHD5 and ATGL might shuttle between nucleus and cytoplasm depending on their lipid droplet association. It also raises the questions of a possible function of the nuclear ATGL and ABHD5 pools and of the nature of the signals mediating the protein translocation. Altogether, as for ABHD5 [30], ATGL capacity to degrade lipid droplets correlated with its pro-viral function (Fig 7E).

### ABHD5 and ATGL also participate in hepatic lipoprotein secretion

The HCV lipo-viro-particle and lipoproteins share a common density range, a neutral lipid core and apolipoproteins at their surface, the latter being essential for their biogenesis (for a review, see [24]). We hypothesized that HCV and the host lipoproteins rely on the same lipolytic machinery to extract the triglycerides from the lipid droplets and to build up their inner neutral lipid core. To verify that this was the case in our HCV-permissive cell line, we titrated the secreted ApoB and E upon manipulation of ABHD5 or ATGL expression (S6 Fig). Globally, ApoB secretion correlated with ABHD5 and ATGL expression levels (S6A Fig). Both ABHD5 and ATGL knockdown reduced extracellular ApoB levels. This effect was rescued by ectopically expressing a RNAi-resistant but functional form of the protein but not with a non-functional mutant. On the contrary, ApoE production and secretion were not significantly modified upon ATGL or ABHD5 manipulation (S6B Fig). In conclusion, ABHD5 and ATGL likely participate in hepatic lipoprotein secretion, as well as HCV production, although they are dispensable for ApoE secretion in our system.

### ABHD5 and ATGL interact and cooperate for HCV production and hepatic lipid droplet lipolysis

**Fig 9:**
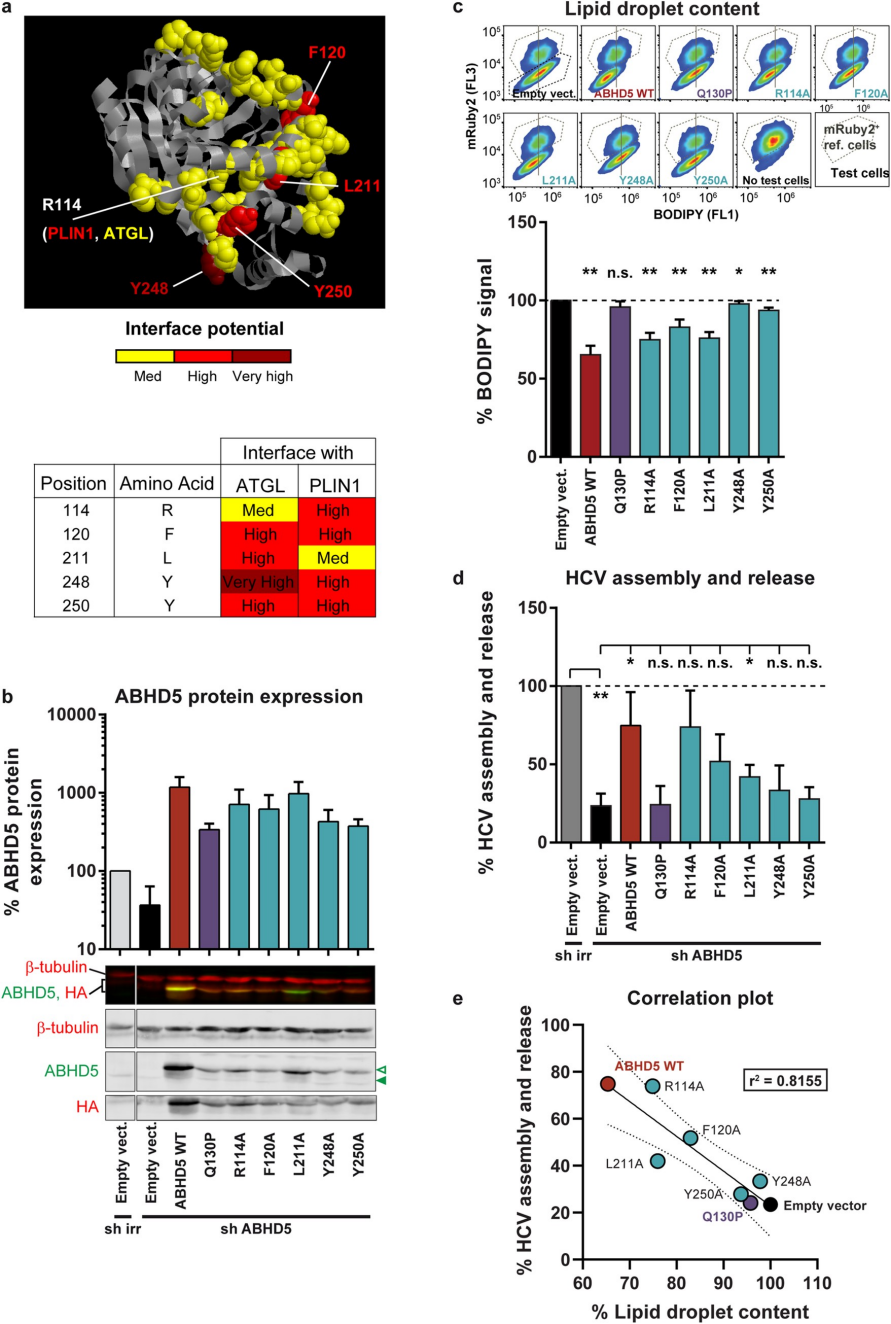
ABHD5 mutants of the predicted ATGL interface have impaired co-lipase and pro-viral functions. (**a**) ABHD5 interaction to ATGL was predicted using Interactome INSIDER (http://interactomeinsider.yulab.org/, [46]) and the involved residues were depicted on the ABHD5 3D model (ModBase B2R9K0, ribbon representation with key residues as space-filling spheres). We mutated those residues that belong with a high or very high confidence to the ATGL and/or PLIN1 interface. Note that the two predicted interfaces overlap, but R114 has a higher interface potential with PLIN1 while L211 is more likely to interact with ATGL. (**b**) Verification by Western blot of ABHD5 protein expression upon knockdown and complementation in Lunet N hCD81 FLuc cells, 4 days post lentiviral transduction (at the time when HCV replication and production were assessed). Protein expression was quantified with the Odyssey imager and normalized to β-tubulin and to ABHD5 endogenous expression level (n = 3). Green arrowheads indicate the HA-tagged (empty arrowhead) and endogenous (full arrowhead) ABHD5 proteins. (**c**) Cellular lipid droplet content in Lunet N hCD81 cells upon expression of the different ABHD5 mutants. Representative flow cytometry plots are shown at the top and the average effects are plotted at the bottom (n = 5). (**d**) HCV assembly and release in Lunet N hCD81 FLuc cells upon ABHD5 knockdown and complementation with shRNA-resistant ABHD5 mutants of the predicted ATGL/PLIN1 interface (n = 3). (**e**) Correlation between HCV production and lipid droplet lipolysis. This graph gathers the results plotted in panels c and d as well as the linear regression (full line), 95% confidence band (dotted lines) and r^2^ value. Note that the lipid droplet content is tested in an over-expression assay (X axis), whereas HCV assembly and release are assessed in the context of the rescue of an ABHD5 knockdown (Y axis). This is why the empty vector is set to 100% on the X axis, but to a lower value for the Y axis (presence of ABHD5 shRNA).

**Fig 10:**
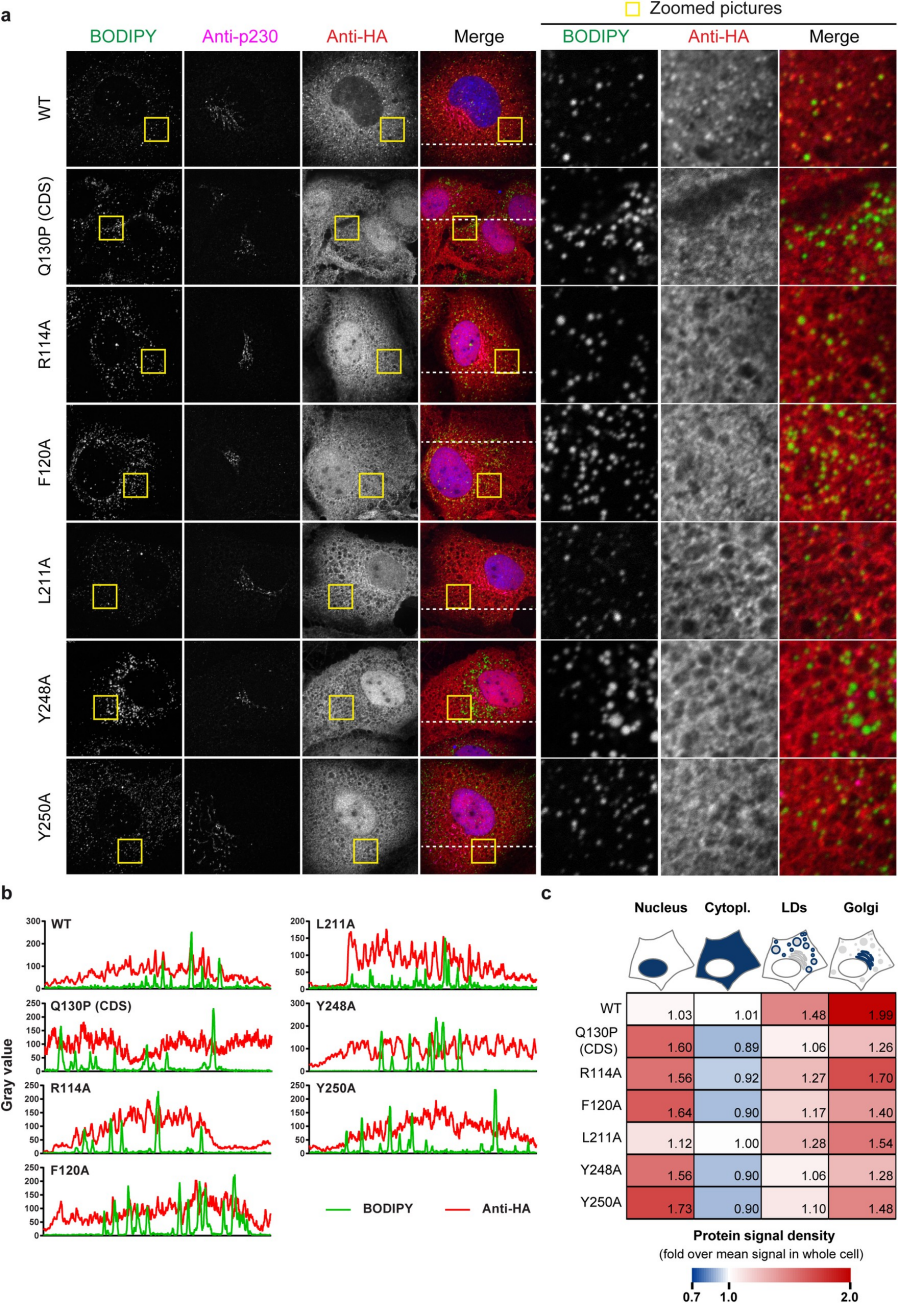
Subcellular localization of the ABHD5 mutants of the predicted ATGL interface. (**a**) Immunofluorescence analysis of HA-tagged ABHD5 localization respectively to the lipid droplets (BODIPY 493/503) and *trans*-Golgi (p230 marker) in transduced Lunet N hCD81 cells. (**b**) Intensity profiles of the green and red channels along the line depicted in the merge pictures of panel a. (**c**) Quantification of ABHD5 accumulation in the nucleus, the cytoplasm, the Golgi apparatus and at the lipid droplet periphery. The heatmap shows the fold enrichment of the protein signal in each compartment as compared to the mean protein signal in the whole cell. The average of 3 experiments with at least 10 pictures per experiment is depicted.

**Fig 11:**
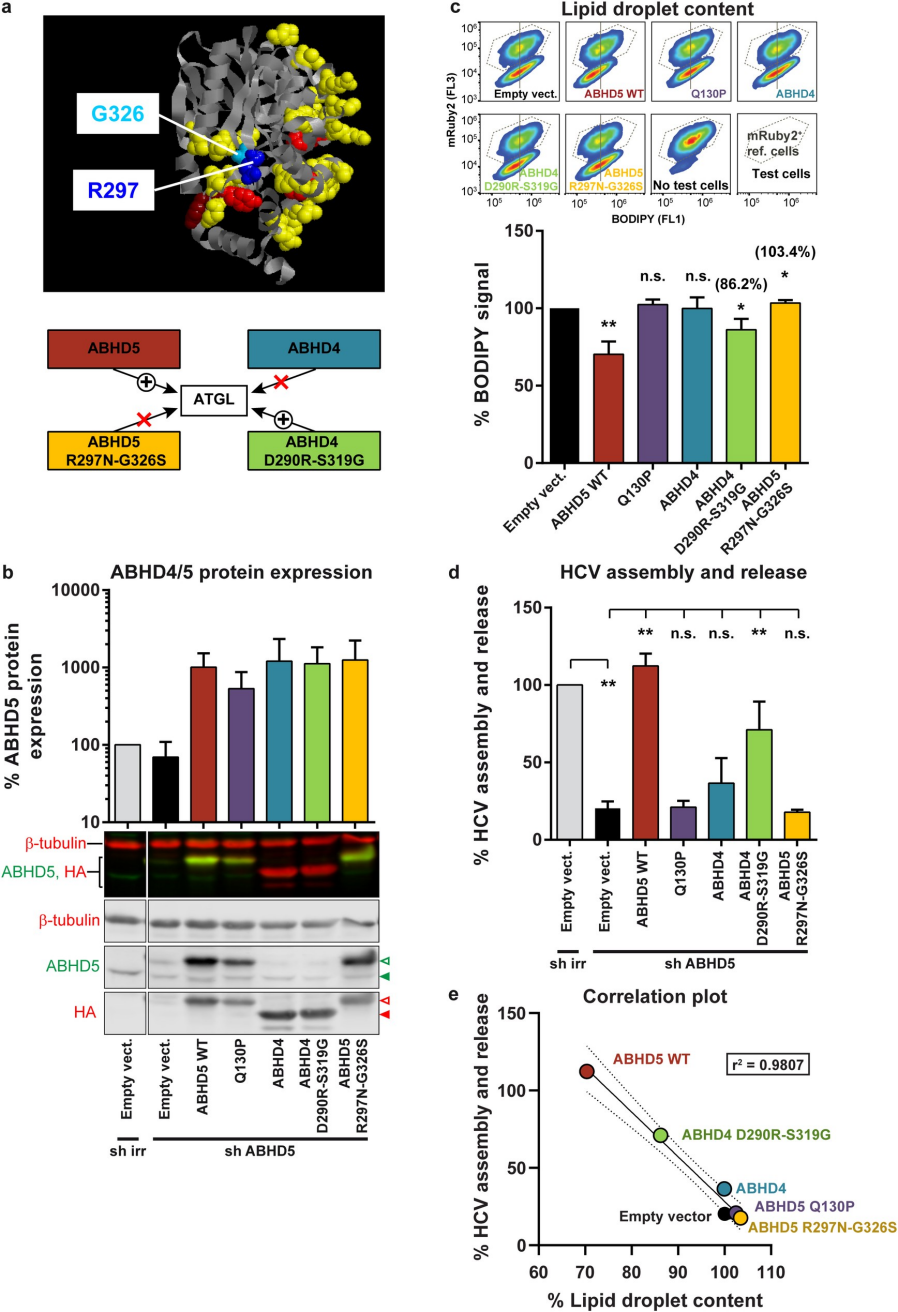
Transfer of the lipolytic and pro-viral functions by swapping two residues between ABHD5 and its paralog ABHD4. (**a**) Swapping two residues between ABHD4 and ABHD5 confers ATGL activation properties to ABHD4 and results in a loss-of-function for ABHD5. ABHD4/5 chimeras were designed based on the results obtained with the murine homologs by Sanders and colleagues [47]. The human ABHD4 D290R-S319G mutant was also shown not to activate ATGL [47]. Here we further engineered a loss-of-function human ABHD5 mutant encoding the mouse ABHD4 residues instead of the R297 and G326, shown in the ABHD5 mouse homolog to be important for ATGL activation (R299 and G328) [47]. These residues are depicted in blue on ABHD5 predicted 3D model ABHD5 (ModBase B2R9K0) with the Interactome INSIDER predicted ATGL interface in yellow to red (see Fig 9A). (**b**) Verification by Western blot of ABHD4/5 protein expression in Lunet N hCD81 Fluc cells upon knockdown and complementation, 4 days post lentiviral transduction (at the time when HCV replication and production were assessed). Protein expression was quantified with the Odyssey imager and normalized to β-tubulin and to ABHD5 endogenous expression level, directly by comparing the ABHD5 antibody signal intensities or indirectly with the HA antibody signals for those constructs that are not detected by the ABHD5 antibody (n = 3). Green arrowheads indicate the HA-tagged (empty arrowhead) and endogenous (full arrowhead) ABHD5 proteins. Red arrowheads indicate the HA-tagged ABHD5 (empty arrowhead) and ABHD4 (full arrowhead) proteins. Note that ABHD5 has 349 residues whereas ABHD4 is 7 amino acids shorter, hence the size difference. (**c**) Cellular lipid droplet content in Lunet N hCD81 cells upon expression of the different ABHD4/5 mutants. Representative flow cytometry plots are shown at the top and the average effects are plotted at the bottom (n = 5). (**d**) HCV assembly and release in Lunet N hCD81 Fluc cells upon ABHD5 knockdown and complementation with shRNA-resistant ABHD4/5 mutants (n = 4). (**e**) Correlation between HCV production and lipid droplet lipolysis. This graph gathers the results plotted in panels c and d as well as the linear regression (full line), 95% confidence band (dotted lines) and r^2^ value. Note that the lipid droplet content is tested in an over-expression assay (X axis), whereas HCV assembly and release are assessed in the context of the rescue of an ABHD5 knockdown (Y axis). This is why the empty vector is set to 100% on the X axis, but to a lower value for the Y axis (presence of ABHD5 shRNA).

**Fig 12:**
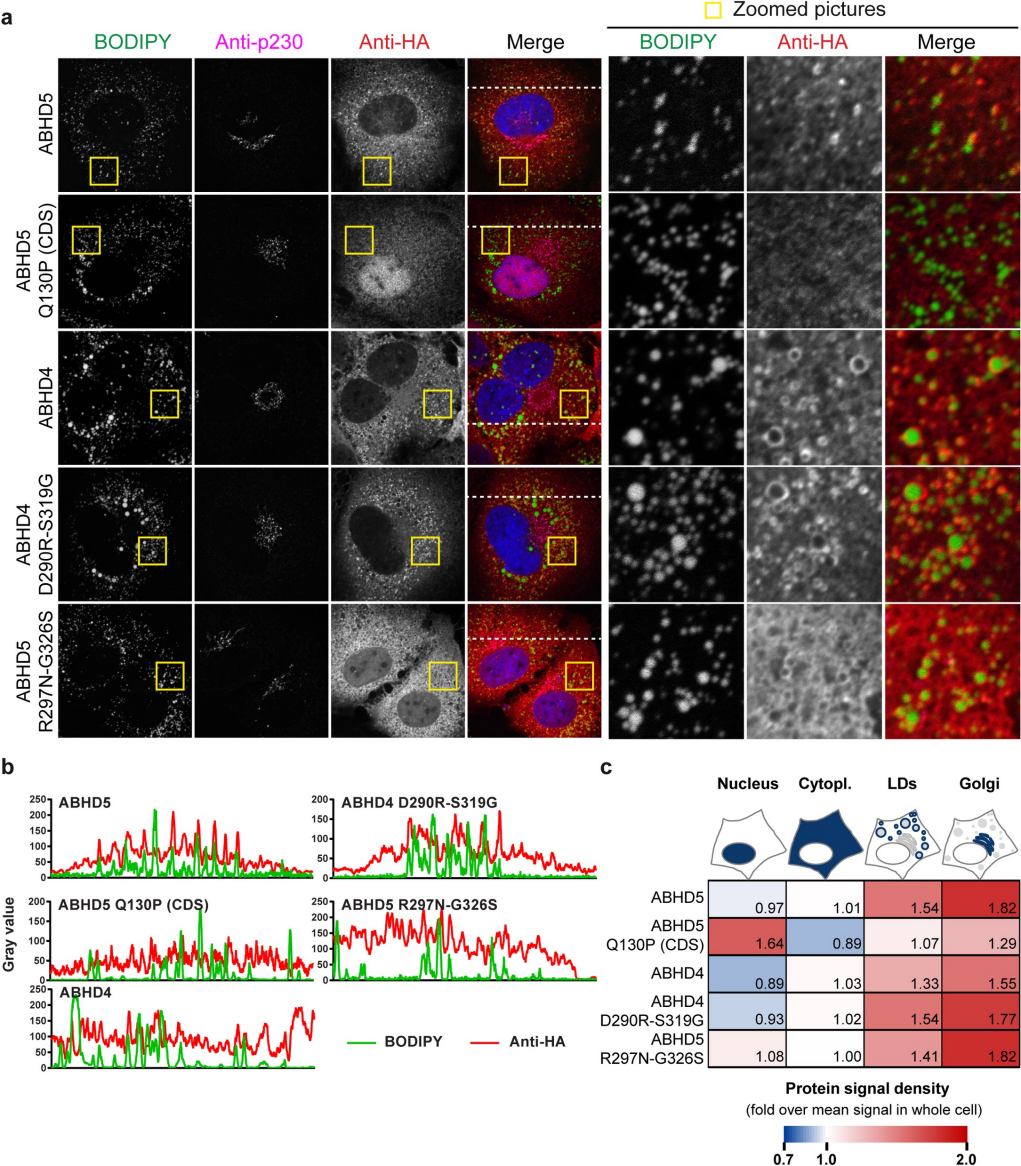
Subcellular localization of the ABHD4/5 mutants. (**a**) Immunofluorescence analysis of HA-tagged ABHD4/5 localization respectively to the lipid droplets (BODIPY 493/503) and *trans*-Golgi (p230 marker) in transduced Lunet N hCD81 cells. (**b**) Intensity profiles of the green and red channels along the line depicted in the merge pictures of panel a. (**c**) Quantification of ABHD4/5 accumulation in the nucleus, the cytoplasm, the Golgi apparatus and at the lipid droplet periphery. The heatmap shows the fold enrichment of the protein signal in each compartment as compared to the mean protein signal in the whole cell. The average of 3 experiments with at least 10 pictures per experiment is depicted.

To confirm that ABHD5 and ATGL cooperate for HCV assembly, we aimed at specifically disrupting ABHD5´s capacity to bind and activate ATGL. To this end, we took advantage of the Interactome INSIDER protein interface prediction tool [46] and mutated the presumed ABHD5 interaction interface to ATGL (Figs 9 and 10). In addition, we created chimeras between ABHD5 and its paralog ABHD4. Because ABHD4 is unable to activate ATGL [47], we examined whether amino acid-swaps between the paralogs transfer the ability to activate ATGL for lipid droplet lipolysis and HCV assembly in our hepatoma cells (Figs 11 and 12).

The Interactome INSIDER [46] references two interactors for ABHD5: ATGL and PLIN1. Note that PLIN1 is mostly expressed in adipocytes [1] (0.13 RPKM in our set of PHHs from 3 donors, NCBI database, GEO accession number GSE132548, (Tegtmeyer B, Vieyres G, submitted for publication)) and therefore this interaction is unlikely to be relevant in our study system. The predicted interaction interfaces were overlapping, with 5 residues having a high or very high interface potential for one or the other protein (R114, F120, L211, Y248 and Y250), including 3 common residues (F120, Y248 and Y250) (Fig 9A). Alanine mutants of these residues were readily expressed (Fig 9B) and mutation of either tyrosine residue reduced both ABHD5 lipolytic function and abolished its pro-viral activity (Fig 9C and 9D, S7 Fig). Mutations of the other residues of the predicted interface resulted in a milder loss of function (Fig 9C and 9D). Overall, lipolytic and pro-viral activities correlated over the tested mutants (Fig 9E), even though mutant F120A was lipolytic (Fig 9C) but did not significantly boost HCV production (Fig 9D, p-value = 0.09). Importantly, these single point mutations strongly affected ABHD5 subcellular localization (Fig 10 and S8 Fig). Comparably to the Q130P Chanarin-Dorfman syndrome mutant, most mutants were abnormally enriched in the cell nucleus (Fig 10A and 10C) and poorly associated with the lipid droplets (Fig 10A and 10B, S8A and S8B Fig). Indeed, while the signal of WT ABHD5 was 48% denser at the lipid droplet surface as compared to the average signal density in the cell, this concentration factor dropped to 6–28% for the mutants (Fig 10C). While oleic acid stimulation of lipid droplet growth partially rescued the cytoplasmic localization of the R114A, F120A and L211A mutants, all mutants remained less enriched at the lipid droplet surface than the WT (S8C Fig). Taken together, these data suggest that disruption of the ABHD5 interface region for contact to ATGL disturbs functioning of ABHD5 in lipid droplet lipolysis, and HCV assembly. Moreover, the interface mutations also affect subcellular localization of ABHD5.

To complement this data, we used another approach to disrupt the possible cooperation between ABHD5 and ATGL. ABHD4 is ABHD5´s closest paralog [48]. Although these two proteins diverged around 500 million years ago, a sequence identity between 50–55% still suggests some relationship between these proteins (S7 Fig). Functionally however, the two proteins are diverse and ABHD4 is unable to activate ATGL [47]. Sanders and colleagues generated chimeras between the two paralogs and identified two key residues in ABHD5 responsible for its unique functions, namely R299 and G328 in mouse ABHD5 [47] (S9 Fig). Switching these two residues between the two proteins results in a loss-of-function for ABHD5 and a gain-of-function for ABHD4, in regards to ATGL activation [47]. We constructed the corresponding human mutants, ABHD4 D290R-S319G (also tested in [47]) and ABHD5 R297N-G326S (carrying the equivalent mouse ABHD4 residues, known not to activate ATGL) (Fig 11A and S9 Fig). All proteins were expressed and detected with the anti-ABHD5 or anti-HA antibody (Fig 11B). Consistently with the results by Sanders and colleagues [47], ABHD4 was unable to degrade lipid droplets, but the two mutations (ABHD4 D290R-S319G) partially conferred a pro-lipolytic function to the engineered protein (Fig 11C). Consistently, ABHD4 did not rescue HCV production in an ABHD5 knockdown setup, but the double mutant ABHD4 D290R-S319G did (Fig 11D and S10 Fig). On the opposite, the loss-of-function ABHD5 mutant (ABHD5 R297N-G326S) could neither induce lipolysis (Fig 11C) nor support HCV production (Fig 11D). Contrary to the interface mutants, permuting these two residues between ABHD4 and 5 did not affect the protein subcellular localization (Fig 12 and S11 Fig) suggesting that gain of function / loss of function was independent of altered subcellular localization. ABHD4 as well as the chimeric ABHD4/5 mutants were mostly in the cytoplasm and accumulated efficiently at the lipid droplet surface (Fig 12 and S11 Fig). Importantly, over the two tested mutant panels, the lipolytic and pro-viral functions of ABHD5 correlated (Figs 9E and 11E). Altogether, these results suggest that ABHD5 main function in HCV morphogenesis is ATGL activation. The ABHD5 R297N-G326S mutant and the tribasic lipid droplet consumption motif (TBLC) mutant (KRK233-235AAA) that we previously described [30] are to our knowledge the only mutants where ABHD5 function is abrogated but ABHD5 localization is conserved.

**Fig 13:**
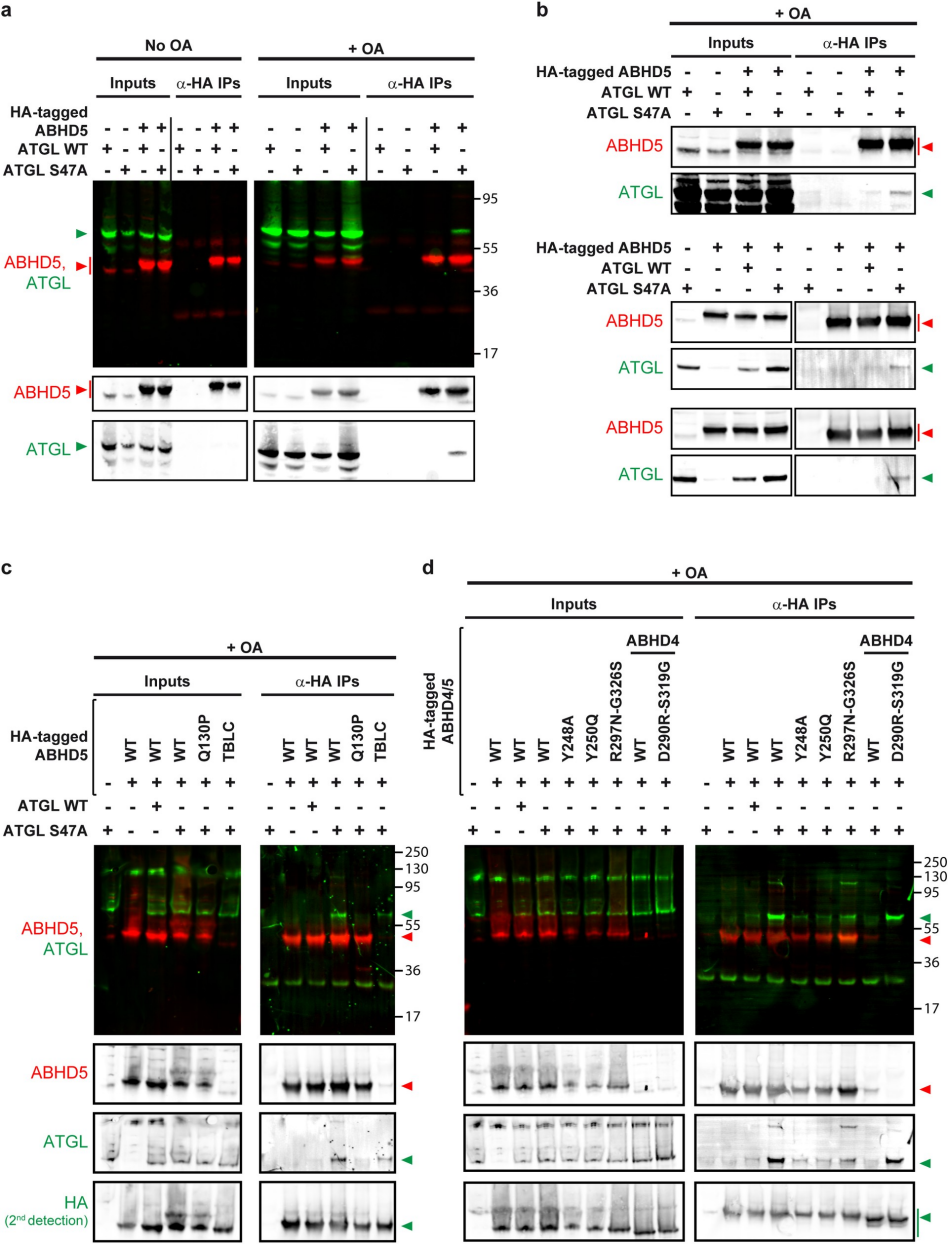
Interaction between ABHD5 and ATGL depends on ABHD5 residues R297 and G326. We transduced Lunet N hCD81 cells to co-express HA-tagged ABHD5 constructs and ATGL (WT or S47A) and harvested the cell lysates 48 hours post-transduction. We incubated the lysates on an anti-HA resin and detected ABHD5 and ATGL with specific antibodies by Western blot (mouse anti-ABHD5 and rabbit anti-ABHD5) in the inputs and the eluates. Note that roughly 5% of the inputs were loaded on the gels. (**a**) Immunoprecipitation of ATGL and ATGL S47A with HA-tagged ABHD5, with or without oleic acid induction of the cells. When indicated, we treated the cells overnight with 100 μM oleic acid before harvesting the lysates. (**b**) Three more examples depicting the co-immunoprecipitation of ATGL S47A together with HA-tagged ABHD5, in oleic acid-treated cells, in different experiments. Note that in two additional experiments, the interaction was under the detection limit. (**c**) Co-immunoprecipitation assay with the Chanarin-Dorfman syndrome mutant Q130P and the ABHD5 TBLC mutant. (**d**) Co-immunoprecipitation assay with the ABHD5 mutants of the predicted ATGL interface or with the ABHD4/5 chimeras. (**c**, **d**) Since the TBLC mutant and the ABHD4 constructs are not recognized by the anti-ABHD5 antibodies ([30] and Fig 11B), we also stained the membranes with an anti-HA antibody (rabbit) after the initial detections with the anti-ABHD5 and anti-ATGL antibodies.

Having identified several residues of ABHD5 essential for its pro-viral function and for ATGL activation, we wanted to verify that these residues also impact on the physical interaction between the lipase and its co-factor. We co-expressed HA-tagged ABHD5 and ATGL in Lunet N hCD81 cells and immunoprecipitated ABHD5 with an anti-HA antibody. In normal conditions, we failed to detect an interaction between lipase and co-lipase (Fig 13A, left panel). The ATGL S47A catalytic site mutant however co-precipitated with ABHD5 when the cells were treated with oleic acid (Fig 13A, right panel). This interaction was reproducible, although the quantity of ATGL S47A co-precipitating with HA-ABHD5 varied depending on the experiment (see eluates for the samples over-expressing WT HA-tagged ABHD5 and ATGL S47A in Fig 13A, 13B, 13C and 13D). Using the ATGL S47A mutant and the oleic acid treatment, we then tested a selection of our ABHD5 mutants. The Chanarin-Dorfman syndrome Q130P mutant is both non-functional and mislocalized [30]. We could not detect its interaction with ATGL S47A (Fig 13C). We previously described the TBLC mutant (KRK233-235AAA) as equally non-functional but properly localized [30]. Interestingly, this mutant could still pull down ATGL S47A, suggesting that it might be defective in ATGL activation rather than binding (Fig 13C). We also tested our presumed ABHD5 interface mutants (Fig 13D). Interestingly, mutation of the tyrosine residues predicted in the ABHD5/ATGL interface (Fig 9) decreased ABHD5 interaction with ATGL S47A. Furthermore, ABHD4 did not pull down ATGL S47A but swapping residues between ABHD4 and ABHD5 transferred the capacity to interact with the lipase, consistently with the functional assays (Fig 13D). Altogether, our results support a physical interaction between ABHD5 and ATGL in hepatic cells and point at several residues involved. The two tyrosines Y248 and Y250 as well as the glutamine residue Q130 might be involved indirectly, as their mutation also affects the protein subcellular localization. However, our data indicate that R297 and G326 are key residues for the lipase / co-lipase interaction, and therefore for the role of ABHD5 in HCV production.

## Discussion

In summary, we identified the ATGL lipase as a new host factor for HCV assembly and release and with it further unravelled the link between hepatic lipid droplet hydrolysis and viral morphogenesis. Indeed, as for ABHD5 (Figs 9E, 11E and [30]), ATGL pro-lipolytic activity correlated with its pro-viral function (Figs 4D and 7E). Furthermore, we could demonstrate that ATGL and ABHD5 worked as a tandem in HCV production. First, the two proteins colocalized (Fig 3) and interacted (Fig 13) in hepatoma cells. Importantly, we had to use the catalytic site inactive mutant of ATGL (S47A) and to induce the cells with oleic acid in order to detect this interaction (Fig 13). Similarly, the colocalization between ABHD5 and ATGL was stronger when using the ATGL S47A mutant and oleic acid treatment (Fig 3). In absence of oleic acid treatment and when co-expressing functional lipase and co-lipase, we observed that most lipid droplets are degraded and an important fraction of the ABHD5 and ATGL pools are diffuse in the cytoplasm, with only few colocalization events on some lipid droplet remnants. It is likely that a minimal cellular lipid droplet organelle volume is necessary as a platform for the interaction to occur and to be captured in detectable amounts by co-immunoprecipitation. Preventing excessive lipolysis by using the S47A mutant rather than WT ATGL and compensating lipid droplet degradation (catalysed by the endogenous ATGL and boosted by over-expressed ABHD5) by treating the cells with oleic acid likely helped overcoming these limitations in our assay.

Secondly, mutation of the predicted ABHD5 interface to ATGL abrogated ABHD5 pro-viral activity (Fig 9). These loss-of-function ABHD5 mutants were mislocalized (Fig 10 and S8 Fig). An explanation for this aberrant localization could be that ATGL interaction is necessary for ABHD5 lipid droplet association. However, ABHD5 can directly bind the lipid droplets via its N-terminal helix [49] and can also interact with several perilipins, including PLIN1, PLIN2 and PLIN5 [33]. Conversely, ABHD5 variants were described that are able to bind ATGL but incapable of associating with the lipid droplets, suggesting that ATGL binding is not the driving force for ABHD5 localization [33]. Therefore, a more likely explanation is that the targeted residues might play an indirect role in ABHD5 function by ensuring the protein proper folding or the exposure of the N-terminal lipid-droplet-binding domain. As an alternative way to abrogate the ABHD5-ATGL cooperation, we made use of ABHD4, the closest paralog of ABHD5, which is unable to activate ATGL [47]. Swapping two residues between ABHD4 and ABHD5 determined both the protein capacity to degrade the lipid droplets and support HCV assembly (Fig 11), without affecting the protein subcellular localization (Fig 12 and S8 Fig). This indicates that the main role played by ABHD5 in HCV production is indeed the activation of the ATGL lipase. Thereby, we believe that the mechanism of ABHD5 involvement in HCV production (both the assembly and release of the lipo-viro-particle [30]) will also apply to ATGL.

In addition to this, our results indicate that pathogenic ATGL mutants are not able to support HCV production (Fig 7). As for ABHD5, the rarity of ATGL minor alleles precludes epidemiological studies [50]. This suggests however that ABHD5 and ATGL might co-determine HCV infection outcome *in vivo*. Interestingly, some of the ATGL NLSDM mutants but also the phosphomimetics T370D mutant were mislocalized: they concentrated in the nucleus instead of associating with the lipid droplets (Fig 8). Similarly, most non-functional natural or engineered ABHD5 single point mutants that we investigated so far, poorly associated with the lipid droplet surface and instead accumulated in the cell nucleus (Fig 10, S8 Fig and [30]). This suggests that ATGL and ABHD5 can shuttle between a cytoplasmic, mostly lipid droplet-associated, and a nuclear protein pool. The shuttling of ATGL for degradation in the nucleus was previously reported [51] but to our knowledge, the mechanism supporting ABHD5 translocation to the nucleus is unknown. The possible nuclear function of these two proteins would be worth investigating, especially in light of the blooming literature on nuclear lipid droplets (see for instance [52, 53]).

Altogether, with the ATGL lipase and its ABHD5 co-factor, we identified a host lipolytic pathway participating in the morphogenesis of the HCV lipo-viro-particle. While ATGL is the main triglyceride lipase [36] and a known ABHD5 effector in adipocytes [34], its contribution to ABHD5´s lipolytic pathway in hepatocytes has been debated [33, 54]. First, only low expression levels had been reported for ATGL in the liver. Indeed, at the mRNA level, ATGL was detected in the Genotype-Tissue Expression (GTEx) RNA-Seq project with 30 RPKM in the liver, against 403 RPKM on average in the adipose tissue (as referenced in the Human Protein Atlas, https://www.proteinatlas.org/ [55]). At the protein level, in human, ATGL was below the detection cut-off in some proteomics studies, e.g. [56], while in another study in mice, ATGL protein expression level was 640 times lower in the liver as compared to the brown adipose tissue [57] (both studies are referenced in the EMBL-EBI Expression Atlas, https://www.ebi.ac.uk/gxa/home [58]). In our set of PHHs, ATGL was found with 6,6 RPKM and ABHD5 with 17,7 RPKM (NCBI database, GEO accession number GSE132548, (Tegtmeyer B, Vieyres G, submitted for publication)). As landmarks, in the same dataset, the essential HCV host assembly factors PI4KIIIα and Occludin were expressed at 6.4 and 2.7 RPKM, respectively, highlighting that even host factors with low mRNA expression might play crucial roles in HCV replication cycle. Furthermore, we could confirm ATGL protein expression by Western blot in various hepatic cell lines as well as in PHHs (Fig 1E).

Secondly, ABHD5 and ATGL pathogenic variants are both associated with neutral lipid storage diseases affecting multiple organs, but with a different organ spectrum [50, 54]. Specifically, ABHD5 mutations cause the Chanarin-Dorfman Syndrome, which is always associated with ichthyosis, a condition that is not observed with the ATGL mutations [50]. Importantly, liver manifestations are more common with ABHD5 mutations than with ATGL mutations. In fact, 80% of the 129 reported Chanarin-Dorfman syndrome patients present liver dysfunction, which can range from hepatomegaly to liver steatosis or cirrhosis. Moreover, monoallelic ABHD5 mutations already predispose to non-alcoholic fatty liver disease [59]. On the other hand, over the 55 patients reported worldwide who carry two pathogenic ATGL alleles, only 20% suffer from liver dysfunction, which is usually limited to hepatomegaly. This evidence suggested that ABHD5 and ATGL have overlapping as well as specific roles that are tissue-dependent, and that ATGL is not the only ABHD5 effector in the liver. Consistently, in the skin, ABHD5 stimulates PNPLA1 for the synthesis of epidermal lipids that are crucial for the skin barrier function [60], and this activity is completely independent of ATGL [50]. In the liver, contrasting results have been reported. In genetic mouse models, targeting ABHD5 or ATGL both increased the liver triglyceride content, but only ABHD5 affected the serum very low-density lipoprotein (VLDL)-associated triglyceride levels [61–63]. Furthermore, in the mouse liver, ABHD5 seems to influence the triglyceride metabolism both in an ATGL-dependent and an ATGL-independent manner, and ABHD5 knockdown causes hepatic steatosis even in the genetic absence of ATGL [64]. These data support the notion that the liver expresses another lipase that may cooperate with ABHD5. Of note, a recent article described that ABHD5 also acts as a serine protease and cleaves the histone deacetylase 4 (HDAC4) with cardioprotective effects [65]. It is unlikely that the cleavage of HDAC4 contributes to the pro-viral effect of ABHD5 we reported. Indeed, residues N153, S298 and H327 are predicted to constitute the catalytic protease triad [65] but we observed in our previous work that both mutations N153S and H327A were tolerated for ABHD5 pro-lipolytic and pro-viral activities [30].

The last decade has brought new evidence for a role for ATGL in the hepatic lipid metabolism (for a review, see [37]). However, if ATGL plays an important role in the hepatic lipid droplet degradation, the degraded triglycerides seem to be channelled towards fatty acid oxidation rather than VLDL-dependent triglyceride secretion [62, 63]. Interestingly, in the intestine, ABHD5 and ATGL also participate in lipid droplet degradation but not in chylomicron synthesis as their double knockout in the small intestine of mice does not affect plasma triglyceride levels [66]. Our results confirmed the role of ATGL in lipid droplet degradation in liver-derived cells. Indeed, both the inhibition of endogenous ATGL activity by expressing the G0S2 peptide inhibitor and the knockdown of ATGL lead to lipid droplet accumulation (Figs 4C and 5E). We also formally verified that ABHD5 and ATGL worked as a pair, since variants of ABHD5 incapable of activating ATGL are not functional (Figs 9 and 11). Furthermore, we found that this lipolytic pathway mediated by ATGL and amplified by ABHD5 was involved in both the lipoprotein and HCV productions. Interestingly however, contrary to ApoB, ApoE secretion was not sensitive to the modulation of the ABHD5/ATGL pathway (S6 Fig). It is likely that, as suggested by the literature [62, 63], lipoprotein production in our cell line might proceed mostly independently of ATGL. In fact, provision of lipids for lipoprotein secretion might employ complementary pathways and not rely solely on the ATGL lipase. HCV however might have only evolved to exploit a subset of these pathways, including the ABHD5-ATGL axis, explaining why the modulation of ATGL or ABHD5 expression affects HCV but not ApoE secretion. Interestingly, TM6SF2 is another host factor promoting lipid droplet degradation and both HCV and lipoprotein productions [67]. Nevertheless, both the function of the protein and its relationship to ABHD5-induced lipolysis are unknown. Moreover, its localization is restricted to the ER and the Golgi, suggesting the protein is involved in a later stage of the neutral lipid transfer to the virion. Remarkably, other lipid or lipoprotein pathways hijacked by HCV are highly redundant. This is the case of the panel of lipoprotein receptors involved and interchangeable in HCV entry (Scavenger Receptor class B type I (SR-BI), low and very low density lipoprotein receptor (LDLR and VLDLR)) [68] but also of the spectrum of apolipoproteins permitting HCV morphogenesis [25, 26]. Of note, in the course of our studies, we repeatedly observed that the defect in HCV assembly tended to blur in cells having a long-term depletion of ABHD5 or ATGL expression or a complete gene knockout. In our view, this is a further indication that feedback loops are activated and/or redundant lipolytic pathways are involved that restore the lipid droplet homeostasis and in parallel ensure a certain level of HCV production. Unbiased screening for other lipase candidates might give a better overview on the lipolytic pathways involved in HCV morphogenesis. Since HCV production is likely to be more sensitive than lipoprotein biogenesis to the shutdown of individual lipolytic pathways, it is certainly a precious tool to further dissect the lipoprotein biogenesis.

By targeting simultaneously and transiently several lipolytic pathways and their regulators, a more drastic reduction of HCV production might be achieved. This could permit evidencing directly the lipid transfer between lipid droplets and virions but also might help to investigate the effect of triglyceride recruitment on the lipid composition of the lipo-viro-particle, on its atypical density profile and morphology, and on its neutralization by antibodies. Modulating the virion lipoprotein association might indeed impact on vaccine design and could increase the potential of inactivated cell-cultured HCV as a vaccine candidate [69]. Finally, the identification of ATGL as a host factor for HCV morphogenesis sheds new light on the genesis of the atypical HCV lipo-viro-particle and extends the network of interactions between pathogens and lipid droplets. In the future, it would be interesting to assess whether other pathogens relying on the lipid droplet reservoir also hijack the same pathway.

## Methods

### Ethics statement

All tissue donors gave written informed consent for experimental use of clinical data and liver specimen prior to surgery. The protocol was approved by the ethics commission of Hanover Medical School (#252–2008 and #2148–2014).

### Cell and virus culture

The Huh-7.5 [70], Lunet N hCD81 [71], Lunet N hCD81 FLuc [30], HEK 293T (American Type Culture Collection, ATCC CRL-3612) [72], Huh6 [73], HepG2-HFL [74], 293T/mir-122/ApoE and HeLa/mir-122/ApoE cell lines [75] and their culture conditions have been described before. We used the Huh-7.5 cell lines for producing HCV stocks and the HEK 293T cell line for the lentivirus stocks. Except for Fig 1, we used in all other experiments the Lunet N hCD81 (immunofluorescences, expression assays, lipid droplet content assay, co-immunoprecipitations) or Lunet N hCD81 Fluc cells (HCV whole replication cycle assays), unless otherwise stated. We generated the Lunet N hCD81/mRuby2 cell line by lentiviral transduction of the pWPI-Nter-mRuby2-Puro construct and Puromycin selection (Sigma #P8833, 2,5 μg/ml). We cultivated all cell lines in Dulbecco’s modified Eagle’s medium (DMEM; Gibco #41965–039) supplemented with 2 mM L-glutamine (Gibco #25030–024), non-essential amino acids (Gibco #11140–035), 100 U/ml of penicillin, 100 μg/ml of streptomycin (Gibco #15140–122) and 10% foetal calf serum (FCS, Capricorn Scientific #FBS-11A). In addition to this, we kept the cell lines under the relevant selection. In the case of the newly generated Lunet N hCD81/mRuby2 cell line, the selection consisted of 5 μg/ml Blasticidin (Fisher bioreagents #BP2647-100) and 2.5 μg/ml puromycin (as above). Primary human hepatocytes (PHHs, obtained from the department of General, Visceral and Transplant Surgery at the Hannover Medical School, Germany) were isolated from resected liver specimen using a 2-step-perfusion-technique applying 0.05% collagenase P (Roche Diagnostics) as previously reported [76]. Freshly isolated PHH were seeded on collagen-coated coverslips at a concentration of 0.375 x 10^6^ cells/well in 24-well dishes and maintained in Hepatocyte Culture Medium (HCM, Lonza) at 37°C and with 5% CO_2_ until lysis for Western blot. Lentivirus and HCV stocks were produced as described before [30].

### Plasmid constructs

We used the already described Jc1 [77] and JcR2a [78] HCV constructs. The shRNAs were expressed in the pLenti3_U6_ECEP7 expression vector and described in our previous publication (shABHD5.796 and sh irr) [30]. Similarly, we published before the HA-tagged shRNA-resistant ABHD5 expression construct (pWPI-ABHD5-shResist-L-HA-HA-BLR) and its Chanarin-Dorfman syndrome mutant Q130P, as well as the pWPI-ABHD5-shResist-mCitrine-Puro and the pWPI-Nter-mRuby2-Puro constructs [30].

Here, we cloned two additional series of ABHD5 mutants, the predicted interface mutants and the ABHD4/5 constructs. All were tagged at the C-terminus with a double HA tag (YPYDVPDYA, twice) preceded by a linker (GGGGSG). For the pWPI_ABHD4-shResist-L-HAHA_BLR construct, we conserved the protein sequence corresponding to the accession number NM_022060.2 but codon optimized the nucleotide sequence using the IDT webtool and further added 2 silent mutations in shABHD5.796 target sequence (6 mismatches altogether) and a double HA epitope tag with linker at the C-terminus. This sequence was ordered as a minigene (IDT, Leuven, Belgium) and cloned between AscI and SpeI in the pWPI-BLR vector. For the ABHD4 D290R-S319G mutant, we ordered a gBlock (IDT) corresponding to the PflFI-SpeI fragment and replaced this in the pWPI_ABHD4-shResist-L-HAHA_BLR construct. For the pWPI_ABHD5_R297N-G326S_shResist_L-HAHA_BLR, we used 3-fragments ligation with pWPI-BLR (SpeI-AscI), pWPI-ABHD5-shResist-L-HAHA-BLR (AscI-StuI) [30] and a gBlock covering the StuI-SpeI fragment. The Interactome INSIDER mutants were cloned by fusion PCR using the Q5 polymerase (New England Biolabs #M0491), the AscI and NdeI restriction sites and pWPI-ABHD5-shResist-L-HAHA-BLR [30] as a template.

ATGL constructs were either untagged or had an N-terminal double HA tag followed by a linker (as above), and based on the reference sequence NM_020376.4 (CCDS 7718.1). We introduced at least six silent mutations in the seeding sequences of each of the ATGL SMARTpool siRNAs and of the three individual siRNAs (si aTGL a/b/c, see below) to obtain the WT tagged and untagged constructs (pWPI-HAHA-L-ATGL-7siResist-BLR and pWPI-ATGL-7siResist-BLR). Based on these constructs, we introduced the various point mutations (S47A, D166A, P195L, Q250P, Q289X and T370D) by fusion PCR with the Q5 enzyme and the AscI and SpeI restriction sites. Note that during this project, we also cloned a panel of constructs coding for the same ATGL protein sequences but having a codon optimized nucleotide sequence inserted at a different position of the multiple cloning site. Intriguingly, this cloning context has strong deleterious effects on ATGL function (poor lipase and pro-viral activities) despite no detectable change in protein expression or subcellular localization as compared to the constructs described here.

G0S2 (NM_015714.4, CCDS1488) was cloned in the pWPI-Puro vector either untagged or followed by a glycine-serine linker (GGGGSG) and a Flag epitope tag (DYKDDDDK). The Flag-tagged G0S2 coding sequence was ordered as a gBlock and the untagged G0S2 sequence was amplified from this gBlock by PCR. We inserted both sequences in the pWPI-Puro vector between BamHI and MluI.

All constructs were verified by restriction digests and sequencing of the inserts (Microsynth Seqlab, Göttingen, Germany) whenever PCR or synthetic gene fragments were used.

### Antibodies, dyes, chemicals and siRNAs

To induce lipid droplet accumulation, we combined oleic acid (Sigma #O1008) and bovine serum albumin (BSA) (Gibco, #30036–578) as described before [30], and used a final concentration of oleic acid of 360 μM or 100 μM, as indicated. We transfected siRNAs using Lipofectamine RNAi Max (Invitrogen #13778–150) and the manufacturer’s protocol. We used commercial primary antibodies directed against ABHD5 (mouse monoclonal antibody, clone 1F3, Abnova #H00051099-M01, WB 1/1,000), ATGL (rabbit, Cayman #10006409, WB 1/500), β-tubulin (rabbit antibody, Thermo Scientific, clone E.884.5, #M45-15002, WB 1/1,000), p230 (Golgi marker, BD #611281), HCV E2 (CBH23, human, 1,2 ug/ml [79]) and the HA epitope tag (mouse antibody, Covance #MMS-101P or rabbit antibody, Sigma #H6908). For flow cytometry and recognition of HCV-infected cells, we conjugated the anti-NS5A antibody (9E10) directly to the A647 dye using the AlexaFluor 647 Antibody Labelling Kit (ThermoFisher). For Western blots, we also used the HRP-conjugated anti-β-actin antibody (1/25,000) for chemiluminescence detection (Chemostar Imager, Intas) and species-specific IRDye-conjugated secondary antibodies (Li-cor, 1/15,000) for near-infrared fluorescence imaging (Odyssey CLx imager, LI-COR). For immunofluorescence and flow cytometry, we purchased the Alexa-conjugated secondary antibodies (A488, A568 and A647, used 1/1,000) from Life Technologies and the BODIPY 493/503 from Invitrogen (#D3922, used 1/1,000 for microscopy and 1/3,000 for flow cytometry). The blue lipid droplet dye AUTOdot [42] was from Abgent (#SM1000a).

To knock ATGL expression down, we used the siGENOME SMARTpool from ThermoScientific (#M-009003-01-0005), which comprised of four siRNAs with the following target sequences: D-009003-01, GUA AAG AUC AUC CGC AGU U; D-009003-02, GGG CGA GAG UGA CAU CUG U; D-009003-03, UCUAUGAGCUUAAGAACAC and D-009003-04, UCA UUG AGG UAU CUA AAG A. Alternatively, we used the following three individual Silencer Select pre-designed siRNAs purchased from Life Technologies (#4427037): si ATGL a (s32682, GGG CGA GAG UGA CAU CUG UTT); si ATGL b (s32683, CUU UAC UCC UGA GAA CUU UTT) and si ATGL c (s32684, CCU UCA ACC UGG UAA AGA UTT). As non-targeting siRNAs, we used the Silencer Select Negative Control No. 1 (#4390843, siRNA ID s813, UAA CGA CGC GAC GAC GUA Att) or 2 (#4390846, siRNA ID s814, UCG UAA GUA AGC GCA ACC Ctt) from Thermo Scientific. To knockdown ABHD5 and ApoE, we used the same three siRNAs from Ambion as in our previous publication [30]. The sequences of the ABHD5 siRNAs were reported there and the ApoE siRNAs were as follows: (a) GGAGUUGAAGGCCUACAAAtt; (b) CUAGUUUAAUAAAGAUUCAtt; (c) GACAAUCACUGAACGCCGAtt.

### Western blot and quantification

Western blot was performed as described before [30] with the antibodies listed above, and with either chemiluminescence (Chemostar Imager, Intas) or near-infrared fluorescence detection (Odyssey CLx imager, LI-COR). In the last case, we quantified the signal intensities with the instrument software.

### Luciferase activity assays

To assess the *Renilla* (RLuc) or Firefly (FLuc) luciferase activities, we lysed the cells in milliQ water (400 μl per well for 6-well dishes, 150 μl per well for 12- or 24-well dishes, 40 μl per well for 96-well dishes) and froze them at -80°C until luciferase assay. We determined the luciferase activities using our previously published protocols [80, 81].

### Rescue of ABHD5 shRNA-mediated knockdown

At day 1, we seeded 8x10^4^ Lunet N hCD81 cells in 12-well dishes and transduced them overnight simultaneously with the shRNA and the ABHD5-encoding rescue constructs (or the empty pWPI-BLR vector) using the corresponding lentiviruses mixed. We changed the medium at day 2. We then split the cells at day 3 into 12-well dishes to reach confluence at day 6. We used part of the cells to test HCV whole replication cycle and part to verify ABHD5 expression by Western blot. At day 4, we infected the cells with HCV JcR2a (producer cells for HCV whole replication cycle), changed the medium 4 hours later and seeded target Huh-7.5 cells (10^5^ cells/well in 12-well dishes). At day 5, we inoculated the target cells with the supernatants harvested from the producer cells and lysed the producer cells for luciferase activity assay. In parallel, we harvested the pellets of the uninfected cells for verification of ABHD5 expression by Western blot. We changed the medium of the target cells 4 hours post-inoculation and lysed them at day 9 for luciferase assay.

### ATGL siRNA knockdown

To test the pools of siRNAs (Fig 5A and 5B), we electroporated 4x10^6^ Lunet N hCD81 FLuc cells with JcR2a RNA and resuspended them in 20 ml of complete medium without antibiotics. We seeded the cells in 24-well dishes (0,5 ml / well) and transfected them 4 hours later with a total of 7,5 pmol of siRNAs (pools of 3 siRNAs against ABHD5 or ApoE, SMARTpool of 4 siRNAs against ATGL) and Lipofectamine RNAi Max, according to the manufacturer´s instructions. The next day, we exchanged the medium. We lysed the producer cells for luciferase assays and collected the supernatants 72 and 96 hours post-electroporation (h.p.e.) We infected target cells with the supernatants and measure their luciferase activity 72 hours post-infection (h.p.i.) as above. ATGL mRNA and protein expression and the lipid droplet content upon siRNA knockdown were tested in 24-well dishes with 7,5 pmol siRNA per well (Fig 5C–5E). For the effect of individual ATGL siRNAs on HCV whole replication cycle (Fig 5F and 5G), we electroporated Lunet N hCD81 Fluc with JcR2a RNA as above but seeded the cells in 96-well dishes (100 μl /well). We also transfected the siRNAs 4 hours later and used 1,5 pmol siRNA per well in all conditions.

### ATGL / ABHD5 over-expression and effect on HCV production (Fig 4B, left panel)

At day one, we seeded 8x10^4^ Lunet N hCD81 cells per well in 12-well dishes and transduced them overnight with the relevant lentiviruses. At day 2, we exchanged the medium and at day 3, we infected the cells with JcR2a and changed the medium 4 hours later. At day 4, we changed the medium again, 24 hours before the harvesting. At day 5, we lysed the producer cells and collected the supernatants to infect the target cells as above.

### G0S2 over-expression and effect on HCV production (Fig 4B, right panel)

At day one, we seeded 8x10^4^ Lunet N hCD81 cells per well in 12-well dishes and transduced them overnight with the relevant lentiviruses. At day 2, we exchanged the medium and at day 3 split the cells. At day 4, we infected the cells with JcR2a and changed the medium 4 hours later. At day 6, we lysed the producer cells and collected the supernatants to infect the target cells as above.

### Effect of ABHD5 knockdown on Jc1 whole replication cycle

We transduced Lunet N hCD81 cells with ABHD5-targeting or irrelevant shRNAs, split and infected the cells 72 hours later with Jc1 in 12-well dishes. We changed the medium 4 hours post-infection (1 ml /well) and harvested cells and supernatants 48 hours post-infection. We processed the cells for total RNA extraction and qRT-PCR on HCV and GAPDH RNAs, as described below. We used one third of the supernatant for RNA extraction and qRT-PCR to determine the extracellular HCV RNA amounts. We also transferred supernatants on target Huh-7.5 cells seeded one day before (10^4^ cells per well in 96-well dishes), with 6 replicates and a serial dilution of 1/10 or 1/3 depending on the experiment. We fixed these cells in cold methanol (15 min at -20°C) 48 hours post-infection and immunostained them for HCV NS5A using the anti-NS5A 9E10 antibody [82] and the secondary anti-mouse antibody conjugated to Alexa 488. We scanned the plates and quantified the number of infectious foci with the Immunospot Analyzer (Cellular Technology Limited, CTL) and the same acquisition and analysis parameters for all the plates and experiments.

### Rescue of ATGL siRNA-mediated knockdown

At day 1, we seeded 1,3x10^5^ Lunet N hCD81 FLuc cells per well in 6-well dishes. At day 2, we transduced the cells using lentiviruses with the ATGL-expression constructs or the empty vector (pWPI-BLR) and changed the medium 4 hours later. At day 5, we split each well of cells in 24-well dishes and 6-well dishes to reach confluence at day 9. Note that we tested in parallel (i) HCV whole replication cycle (24-well dishes, HCV-infected and siRNA-transfected), (ii) ATGL knockdown efficiency by Western blot (24-well dishes, siRNA-transfected but no HCV infection) and (iii) ATGL rescue construct expression by Western blot (6-well dishes, no HCV infection, no siRNA transfection). At day 7, we infected part of the 24-well dishes with HCV JcR2a (HCV whole replication cycle assay, producer cells). Four hours post-inoculation, we transfected these cells as well as the non-infected cells in 24-well dishes (verification of ATGL knockdown) with 7,5 pmol per well of ATGL or control siRNAs (Silencer Select Negative Control No. 2) using lipofectamine RNAi Max (0,8 ul /well), according to the manufacturer´s instructions. One day later (D8) and 24 hours before stopping the infection, we replaced the medium with 0,5 ml of complete DMEM and seeded Huh-7.5 cells as target cells (3x10^4^ cells/well in 24-well dishes). At day 9, we inoculated the target cells with the supernatants harvested from the producer cells and changed the medium after 4 hours. We also lysed the producer cells for luciferase assay. In parallel, we harvested the cell pellets for the verification of ATGL knockdown and ATGL rescue construct expression. Finally, at day 12, we lysed the target cells as above for luciferase assay.

### Relative quantification of ATGL and HCV mRNA expression by qRT-PCR

We extracted the total RNAs from the cell lysates and the supernatants with the Macherey-Nagel Nucleospin RNA kit (#740955) and the Zymo Research *Quick*-RNA Viral kit (#R1035), respectively. We performed the qRT-PCR as described before [30]. We quantified ATGL and GADPH in duplex with primers and probes purchased from TIB MolBiol (Berlin, Germany). The set of primers and probe for GAPDH detection was as before [30]. For ATGL detection, we used the following reagents: ATGL S 5´-CTCATCCAggCCAATgTCTg-3´, ATGL A 5´-ACCATCCACgTAgCgCAC-3´, ATGL-FAM 5´-6FAM-TCCCCgTgTACTgTgggCTCA—BBQ-3´. We quantified HCV RNA with the primers and probes previously described [83]. Intracellular HCV RNA was quantified in duplex with GAPDH, as before [83]. The extracellular HCV RNA however was quantified alone.

### Immunofluorescence

At day 1, we seeded 2x10^4^ Lunet N hCD81 cells per well in 24-well dishes on coverslips on the morning, and transduced them overnight with the ATGL, ABHD5 and/or G0S2 expression constructs. At day 2 the medium was changed. To trigger lipid droplet accumulation we treated the cells overnight with oleic acid in a concentration of 360 μM and combined with BSA following a previously described protocol [30]. At day 4 we fixed the cells for 10 min in 3% PFA and stored them in PBS until permeabilization and staining, according to a previously reported protocol [30].

For the colocalization study of ABHD5 and ATGL in HCV-infected cells, we seeded 3.5x10^4^ Lunet N hCD81 cells per well in 24-well dishes and transduced them overnight with ATGL-S47A and HA-tagged ABHD5. A medium change was performed on the next day. At day 4, we infected the cells with Jc1 in the morning. 4 hours post-infection, we split the cells in a 1/5 ratio and seeded them in new 24-well dishes on coverslips. At day 7, we added in the morning oleic acid in a concentration of 360 μM combined to BSA. We fixed the cells 6-8 hours later for 10 min in 3% PFA and stored them in PBS until permeabilization and staining as described earlier [30].

We observed the samples with a Nikon Ti-E microscope equipped with a Yokogawa CSU-X1 spinning disc and an EMCCD DU-888 camera from Andor. All depicted pictures were taken with either 60 or 100x Nikon CFI Apochromat TIRF objectives (CFI Apochromat TIRF 60x Oil/ 1.49/ 0,13 and CFI Apochromat TIRF 100x Oil/ 1.49/ 0,12) and an additional 2x magnification lens inside the spinning-disc unit. Alternatively, we used an Olympus IX81 laser-scanning confocal microscope (Olympus Fluoview 1000) with a 100x magnification lens. We then used a Kalman of 3 and acquired the blue (excited at 405 nm) and red (561 nm) channels in a group and the other 2 channels (green and far red, respectively excited at 488 nm and 647 nm) sequentially.

### Image analysis

Microscopy images were analysed using CellProfiler [84] and Fiji [85]. The image montages presented here were done in Fiji, with automatic contrast enhancement of the individual channels. In some cases, we also applied a rolling ball background substraction, with similar parameters for all samples of the figure. Intensity profiles were measured in Fiji and plotted in GraphPad Prism.

To quantify the subcellular enrichment of ABHD5 or ATGL in different cell organelles we used CellProfiler and adapted our previously described pipeline that segments nuclei (from the Dapi channel), lipid droplets (BODIPY channel) and Golgi (p230 channel) and quantifies the protein signal in these different objects [30]. Note that parameters for the object segmentation, particularly for the lipid droplets, had to be adjusted for different experiments, or for the conditions with or without oleic acid, but the same segmentation parameters were kept for all compared conditions (e.g. for WT and all ATGL mutants within one experiment).

### Flow cytometry-based quantification of the lipid droplet content (in absence of HCV infection, as illustrated in S2A and S2B Fig)

To monitor the lipid droplet content of the cells we adapted our previously published protocol as follows [30]. At day 1 in the morning, we seeded 8x10^4^ Lunet N hCD81 FLuc cells per well in 12-well dishes. On the evening, we transduced the cells overnight with ABHD5-/ATGL-/G0S2-/shRNA-expressing constructs or with an empty vector control and we changed the medium the next day. At day 4, we harvested the cells by trypsinization and spiked in a constant number of Lunet N hCD81/mRuby2 cells (for ABHD5 mutants and ATGL mutants; for Fig 4 and the effect of G0S2 and ATGL over-expression, we used cells 4 days post lentiviral transduction with pWPI-Nter-mRuby2-Puro). We immediately fixed the cell mixtures with Fixation Buffer (PBS-1% FCS-0,5% PFA) and stored them at 4°C until staining.

To save siRNAs, we scaled down the experiment testing the effect of ATGL knockdown on the cell lipid droplet content. In this case, at day 1 in the morning, we seeded 5 x10^4^ Lunet N hCD81 FLuc cells per well in 24-well dishes without antibiotics. In the afternoon, we transfected the siRNAs (7,5 pmol siRNA and 0,8 ul Lipofectamine RNAi Max per well) according to the manufacturer´s instructions. At day 2 we performed a medium change. We harvested the cells at 24-48-72 and 96 hours post-transfection by trypsinization and spiked in a constant number of Lunet N hCD81/mRuby2 cells before fixation as above.

For the staining, we first washed once the cells in Wash Buffer (FWB, PBS-1% FCS) (600g, 3 min) and incubated them for 30 min with BODIPY 493/503 diluted 1/3,000 in FWB on ice. We then washed the cells again twice in FWB, resuspended them in FWB and analysed them with the BD Accuri C6 flow cytometer. We excluded cell debris by gating using the FSC-A and SSC-A and monitored the green (BODIPY 493/503) and red (mRuby2) fluorescence of the cells using the FL-1 and FL-3 channels. Colour compensation was performed according to the manufacturer´s instructions.

Note that the spike-in control cells can be readily distinguished from the test cells by their red fluorescence and serve a dual purpose: (i) by comparing the number of test and spike-in cells, we can exclude cell viability issues, (ii) by calculating the ratios of the BODIPY fluorescence between the test and the control cells, we can robustly detect even small variations in the lipid droplet content and avoid possible tube-to-tube variations occurring during staining or fluorescence measurement.

For representation purposes, the pseudocolor plots depicted in Figs 9C and 11C were drawn in FlowJo. For this, we repeated the manual gating and colour compensation on the raw data in FlowJo, however the whole data quantification was performed in the BD Accuri C6 flow cytometer software.

### Flow cytometry-based quantification of the lipid droplet content in HCV-infected cells

To study the lipid droplet lipolysis in HCV-infected cells (Fig 4E and S3D–S3F Fig), we modified the protocol above as follows. We seeded 5x10^4^ Lunet N hCD81 FLuc cells per well in 12-well dishes and transduced them as above. On day 3, we infected the relevant wells with Jc1 and changed the medium 4 hours post-infection. We harvested the cells on day 5, that is 48 hours post-HCV infection and 96 hours post-transduction, and spiked in the Lunet N hCD81/mRuby2 cells as reference as above. We fixed the cells for 10 min on ice in 3% PFA to inactivate HCV, then resuspended them in Fixation Buffer and stored them at 4°C until staining.

For the staining, we first washed the cells once in FWB and permeabilized them in Permeabilization Buffer (PBS-1% FCS-0,1% Saponin) for 20 min on ice. We then incubated them for 30 min with BODIPY 493/503 (1/3,000) and anti-NS5A antibody directly conjugated with Alexa 647 (1/100) in Permeabilization Buffer on ice. Next, we washed the cells again twice in FWB, resuspended them in FWB and analysed them with a Spectral Analyzer (Sony). We excluded cell debris by gating using the FSC-A and SSC-A and the cell doublets by gating using the FSC-A and FSC-H channels. Spectral unmixing was performed according to the manufacturer´s instructions.

### Apolipoprotein ELISAs

We seeded 8x10^4^ Lunet N hCD81 FLuc cells per well in 12-well dishes and transduced them on the same day with lentiviruses expressing the different shRNAs or ATGL/ABHD5 constructs. When relevant, siRNA transfections (15 pmol siRNA per well) were performed 3 hours later and overnight. At day 2 and 3, we exchanged the medium. At day 4, we harvested the cell supernatants. Extracellular ApoB100 and ApoE (MABTECH, Nacka Strand, Sweden) were measured by ELISA following the manufacturer’s instruction and diluting the samples 10 times in PBS- 0.1% BSA-0.05% Tween before quantification.

### Co-immunoprecipitations

To test the interaction between ATGL and ABHD5, we immunopurified HA-tagged ABHD5 (or the various ABHD4 or ABHD5 mutants) by using anti-HA-antibody conjugated agarose beads (Sigma #A2095) and analysed ATGL co-precipitation. Therefore, we seeded Lunet N hCD81 cells in 10 cm-diameter cell culture dishes at day 1 in the morning to reach confluency 48 hours later. We transduced the cells with the HA-tagged ABHD5 variants and ATGL or ATGL-S47A overnight. At day 2, we changed the medium in the morning and induced lipid droplet formation by adding oleic acid in the evening in a final concentration of 100 μM and combined with BSA as described before [30]. At day 3, we harvested the cells by trypsinization, inactivated the trypsin in complete medium, washed the pellets twice in PBS and stored them at -20°C. We lysed the cell pellets for 10 min on ice by using 1% Triton-X100 in PBS (PBS-Triton) supplemented with protease inhibitor (Pierce #A32953). We isolated the post-nuclear extracts by centrifugation and kept an aliquot (“input”) for the SDS-PAGE. The remaining lysates were incubated with the prewashed anti-HA-antibody conjugated beads (25–30 μl per sample) overnight with rotation. On the next day, we washed the beads in PBS-Triton twice shortly, twice with a 5 min incubation before the centrifugation step, and once shortly. Finally, we washed the beads shortly in water and eluted the proteins in Laemmli buffer. We also supplemented the inputs with Laemmli buffer and treated inputs and eluates for 7 min at 70°C before loading them onto NuPAGE 4-12% Bis-Tris-Gels (Thermo Fisher Scientific #NP0321). SDS-PAGE and Western blot were performed as described before [30] and the used antibodies for the detection are indicated in the figure legend.

### Gene ontology and STRING analyses

We filtered the list of metabolic serine hydrolases from the human serine proteases reviewed by Bachovchin *et al*. [32]. The 77 high confidence lipid droplet-associated proteins were retrieved from a proteome analysis of Huh-7 cells by Bersuker *et al*. [38]. Proteins whose abundance at the lipid droplet is enriched or depleted upon HCV infection were extracted from an analysis in Huh-7.5 cells conducted by Rösch *et al*. [39]. The functional protein association network was performed with STRING 11.0 (https://string-db.org/) [86]. Gene ontology enrichment was analysed with PANTHER (http://www.pantherdb.org/) [87, 88].

### ABHD5 3D model

The ABHD5 3D model is from ModBase (B2R9K0) and based on the PDB 3G02 template (Structure of enantioselective mutant of epoxide hydrolase from *Aspergillus niger* generated by directed evolution). We visualized it using Rastop 2.6.4 (https://www.geneinfinity.org/rastop/), a software derived from Rasmol [89] (initially developed by Roger Sayle, currently maintained by Herbert Bernstein). We used the Clustal Omega tool [90] of the EMBL-EBI website (https://www.ebi.ac.uk/Tools/msa/clustalo/) to align ABHD5 and ABHD4 protein sequences.

### Statistics

Unless otherwise stated, we performed the statistics in Excel or GraphPad Prism 8 on the normalized data using heteroscedastic (two-sample assuming unequal variance) t-Tests with two-tailed distribution.

## Supporting information

S1 Fig (Related to Fig 1) Gene Ontology (GO) and STRING analyses of putative lipid droplet lipases. (**a**) GO annotations of the metabolic serine hydrolases and Huh-7 LD-associated proteins (see Fig 1) were retrieved from PANTHER (http://www.pantherdb.org/) [87, 88]. Relevant GO annotations are highlighted in red. (**b**) STRING analysis of the functional association between the metabolic serine hydrolases and the Huh-7 LD-associated proteins (see Fig 1). Disconnected nodes are not displayed. Only experimental and database-derived interactions were kept and a medium confidence score was chosen (0.4). The connection line thickness reflects the interaction confidence score. The nodes associated with the most relevant GO annotations are color-coded (red for biological process “lipid catabolic process”, green for molecular function “lipase activity”, blue for cellular compartment “lipid droplet”). Genes annotated in italics are poorly expressed in PHH (RPKM<0,5). Genes annotated in yellow were found in the Huh-7 lipid droplet proteome [38] and those in teal belong to the metabolic serine hydrolase family [32]. Note that only four genes overlap between these two datasets: LDAH, MGLL, PNPLA2 (a.k.a. ATGL) and PNPLA3 (a.k.a. adiponutrin) (see also Fig 1A). Among those, ATGL and PNPLA3 are the only ones associated with the three selected GO annotations and ATGL is the only direct known STRING functional interactor of ABHD5. ABHD5 is highlighted with a black box.
(TIF)

S2 Fig (Related to Fig 4) Experimental setups to assay the role of ATGL in lipid droplet lipolysis and HCV assembly and release. (**a**) Flow-cytometry-based lipid droplet lipolysis assay: principle and representative flow cytometry plots. We harvested the cells transduced with the different expression constructs (e.g. empty vector (II) or ATGL expression vector (III)) and spiked in a reference cell population that constitutively expresses mRuby2. As a quality control, we also analysed the reference cell population alone (I). We then stained the cell mixtures with the BODIPY lipid droplet dye. The cells of interest and the reference cells can be distinguished in the red channel (FL3, mRuby2, see the two cell population clouds on the 2^nd^ and 3^rd^ plots) and we normalized the BODIPY signal of the cells of interest to the signal of the reference cells. Representative flow cytometry plots are depicted on the right side. The vertical red line highlights the shift of the ATGL-over-expressing cell population towards the left as compared to the reference cell population, indicating a decrease in lipid droplet content (3^rd^ plot). The cell line transduced with an empty vector on the contrary has a similar lipid droplet content as the reference cell line (2^nd^ plot). (**b**) Representative microscopy pictures illustrating the strategy used in (a). The cells were transduced and mixed as in (a) but the cell mixtures were seeded on coverslips 2 days post-transduction and fixed for immunofluorescence one day later (corresponding to harvest time of the cells for flow cytometry in panel a). We stained the samples with BODIPY and Dapi and further detected the HA-tagged ATGL (detected with the anti-HA antibody and a secondary anti-mouse antibody conjugated to A647) to illustrate the ATGL expression in the mRuby2-negative cell populations. We outlined the mRuby2-positive cell population manually with a yellow dotted line. The roman numerals refer to panel a. The contrasts for the Dapi, BODIPY, and mRuby2 channels were automatically enhanced; for the HA channel (which was negative for images I and II), the intensity for all 3 pictures was multiplied 3 times for better visibility. (**c**) Principle of the HCV whole replication cycle assay, as used in Fig 4B, right panel. Cells were lentivirally transduced with the different expression constructs (e.g. empty vector or G0S2 expression vector) and 3 days later infected with the *Renilla* luciferase (RLuc) reporter JcR2a virus [78]. We test the RLuc activity in these producer cells as a measure of HCV entry and replication. We also transfer their supernatant to target cells in order to measure the infectious titre released. To this end, we assess the RLuc activity of the target cells 3 days post-infection. Finally, we deduce the efficiency of HCV assembly and release by normalizing the RLuc activity in the target cells by the RLuc activity in the producer cells. The panel describes the assay as used in Fig 4B, right panel. Please see the Methods section to see variations in the protocol for the other described experiments. Parts of panels a and c were drawn using BioRender (www.biorender.com).
(TIF)

S3 Fig (Related to Fig 4) ATGL proviral effect and comparison of ATGL lipolytic activity in naive, bystander and HCV-infected cells. (**a**, **b**, **c**) Cell viability (**a**), HCV entry and replication (**b**), and HCV assembly and release (**c**) were assessed upon over-expression of ABHD5, ATGL or G0S2 (see Fig 4B). (**a**) Cell viability was assessed by measuring the Firefly luciferase (FLuc) activity in the producer cells. (**b**) HCV entry and replication were determined by measuring the RLuc activity in the producer cells and normalizing for any effect on cell viability (FLuc producer cells). (**c**) HCV assembly and release were assessed by measuring the RLuc activity in the target cells and normalizing for any effect on earlier steps of the replication cycle (RLuc in the producer cells). Note that panel c shows the same data as Fig 4B, but with a logarithmic scale, for consistency within the figure. (**d**) Monitoring of HCV infection rate, in the set of experiments analysed in Fig 4E and S3C Fig (n = 3). About half the cell population was successfully infected as shown by positive NS5A staining at the end of the experiment. (**e**) Gating strategy. Example flow cytometry plots illustrate the different cell populations and gates, with representative data from empty vector-transduced cells. In the left plot, the wells were kept non-infected. In the right plot, the cells were infected with HCV. In both cases, the cells were harvested at the end of the experiment and mixed with mRuby2-expressing reference cells, gated in black. HCV-infected cells are gated in red, the bystander cells in blue and the naive cells in grey, consistently with the colour code in panel c and in Fig 4E. (**f**) Effect of HCV infection status on the lipid droplet content of cells with altered ATGL activity (n = 3). The plot summarizes the same data as depicted in Fig 4E. Similarly as in Fig 4E, the lipid droplet content of the different cell populations was measured and normalized for the reference mRuby2-expressing cell population, to correct for staining or measurement variations. In this graph however, for each condition on the X axis, the data was normalized to the uninfected cells, treated with the control lentivirus (empty vector or irrelevant shRNA). The steatogenic effect of HCV infection is visible by comparing in each condition the red bars (HCV-infected) to the grey bars (naive). The lipid droplet content in the bystander cells is not altered as compared to naive cells.
(TIF)

S4 Fig (Related to Fig 7) HCV replication cycle upon ATGL knockdown and rescue with ATGL mutants. (**a**, **b**, **c**) Cell viability (**a**), HCV entry and replication (**b**), and HCV assembly and release (**c**) were assessed as described in S3A–S3C Fig. Note that panel c shows the same data as Fig 7D, but with a logarithmic scale, for consistency within the figure (n = 8).
(TIF)

S5 Fig (Related to Fig 8) Subcellular localization and lipid droplet association of ATGL variants. Idem Fig 8, but with oleic acid treatment of the cells to induce lipid droplet accumulation.
(TIF)

S6 Fig (Related to Fig 7) ATGL and its co-factor ABHD5 participate in hepatic lipoprotein synthesis. ApoB (**a**) and ApoE (**b**) levels were measured by ELISA in the supernatants of cells with manipulated ABHD5 (left half of the figure) or ATGL (right half of the figure) protein expression (n = 6). Asterisks indicate significant changes as compared to the control (first bar of each graph, in black).
(TIF)

S7 Fig (Related to Fig 9) HCV replication cycle upon ABHD5 knockdown and rescue with the predicted ABHD5 interface mutants. Cell viability (**a**), HCV entry and replication (**b**), and HCV assembly and release (**c**) were assessed as described in S3A–S3C Fig (n = 3). Note that panel c shows the same data as Fig 9D, but with a logarithmic scale, for consistency within the figure.
(TIF)

S8 Fig (Related to Fig 10) Subcellular localization of the mutants of ABHD5’s predicted interface to ATGL. Idem Fig 10, but with oleic acid treatment of the cells to induce lipid droplet accumulation.
(TIF)

S9 Fig (Related to Fig 11) Alignment of mouse and human ABHD4 / ABHD5 protein sequences. Protein sequences were aligned using the Clustal Omega tool [90] of the EMBL-EBI website (https://www.ebi.ac.uk/Tools/msa/clustalo/). The sequences correspond to the UniProt accession numbers Q8TB40 (human ABHD4), Q3U7M5 (mouse ABHD4), Q8WTS1 (human ABHD5) and Q9DBL9 (mouse ABHD5). Key residues are highlighted in colour, with their position relative to the human ABHD5 sequence indicated below. The two residues differing between ABHD4 and ABHD5 and conferring the ATGL co-factor activity [47] are indicated in red. The position of the catalytic serine residue (occupied by an asparagine residue in ABHD5) is shown in blue. The TBLC motif, conserved between ABHD4 and 5, and crucial for ABHD5 co-lipase activity [30] is in green.
(TIF)

S10 Fig (Related to Fig 11) HCV replication cycle upon ABHD5 knockdown and rescue with the ABHD4/5 mutants. Cell viability (**a**), HCV entry and replication (**b**), and HCV assembly and release (**c**) were assessed as described in S3A–S3C Fig (n = 4). Note that panel c shows the same data as Fig 11D, but with a logarithmic scale, for consistency within the figure.
(TIF)

S11 Fig (Related to Fig 12) Subcellular localization of the ABHD4/5 mutants. Idem Fig 12, but with oleic acid treatment of the cells to induce lipid droplet accumulation.
(TIF)
