# Supplementary figures and images for: The ATGL lipase cooperates with ABHD5 to mobilize lipids for hepatitis C virus assembly
Source: PLoS Pathog. 2020 Jun 15;16(6):e1008554. doi: 10.1371/journal.ppat.1008554 (PMC7316345; doi:10.1371/journal.ppat.1008554)

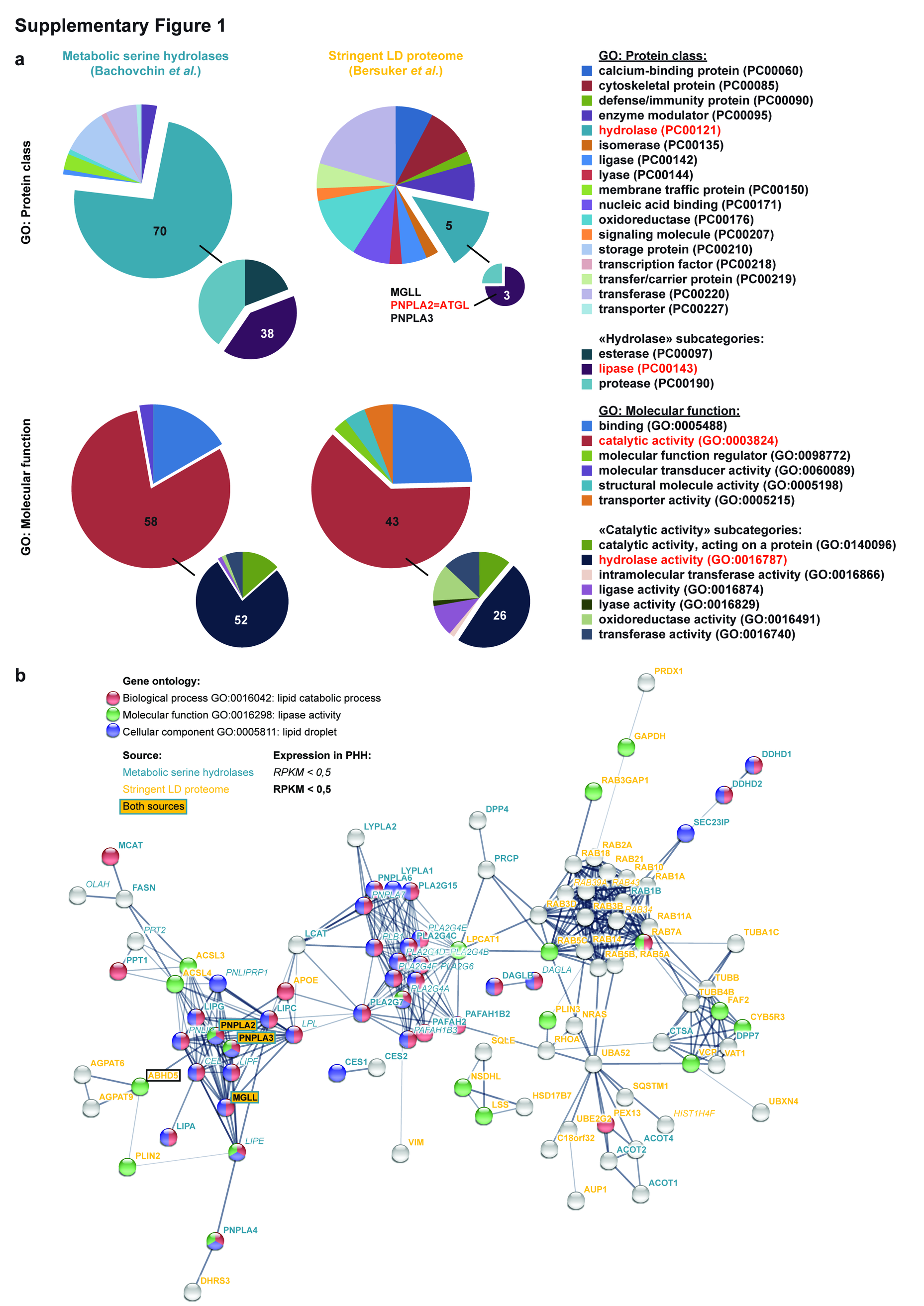

Supplement: S1 Fig — (a) GO annotations of the metabolic serine hydrolases and Huh-7 LD-associated proteins (see Fig 1) were retrieved from PANTHER (http://www.pantherdb.org/) [87, 88]. Relevant GO annotations are highlighted in red. (b) STRING analysis of the functional association between the metabolic serine hydrolases and the Huh-7 LD-associated proteins (see Fig 1). Disconnected nodes are not displayed. Only experimental and database-derived interactions were kept and a medium confidence score was chosen (0.4). The connection line thickness reflects the interaction confidence score. The nodes associated with the most relevant GO annotations are color-coded (red for biological process “lipid catabolic process”, green for molecular function “lipase activity”, blue for cellular compartment “lipid droplet”). Genes annotated in italics are poorly expressed in PHH (RPKM<0,5). Genes annotated in yellow were found in the Huh-7 lipid droplet proteome [38] and those in teal belong to the metabolic serine hydrolase family [32]. Note that only four genes overlap between these two datasets: LDAH, MGLL, PNPLA2 (a.k.a. ATGL) and PNPLA3 (a.k.a. adiponutrin) (see also Fig 1A). Among those, ATGL and PNPLA3 are the only ones associated with the three selected GO annotations and ATGL is the only direct known STRING functional interactor of ABHD5. ABHD5 is highlighted with a black box. (TIF) [file ppat.1008554.s001.tif]

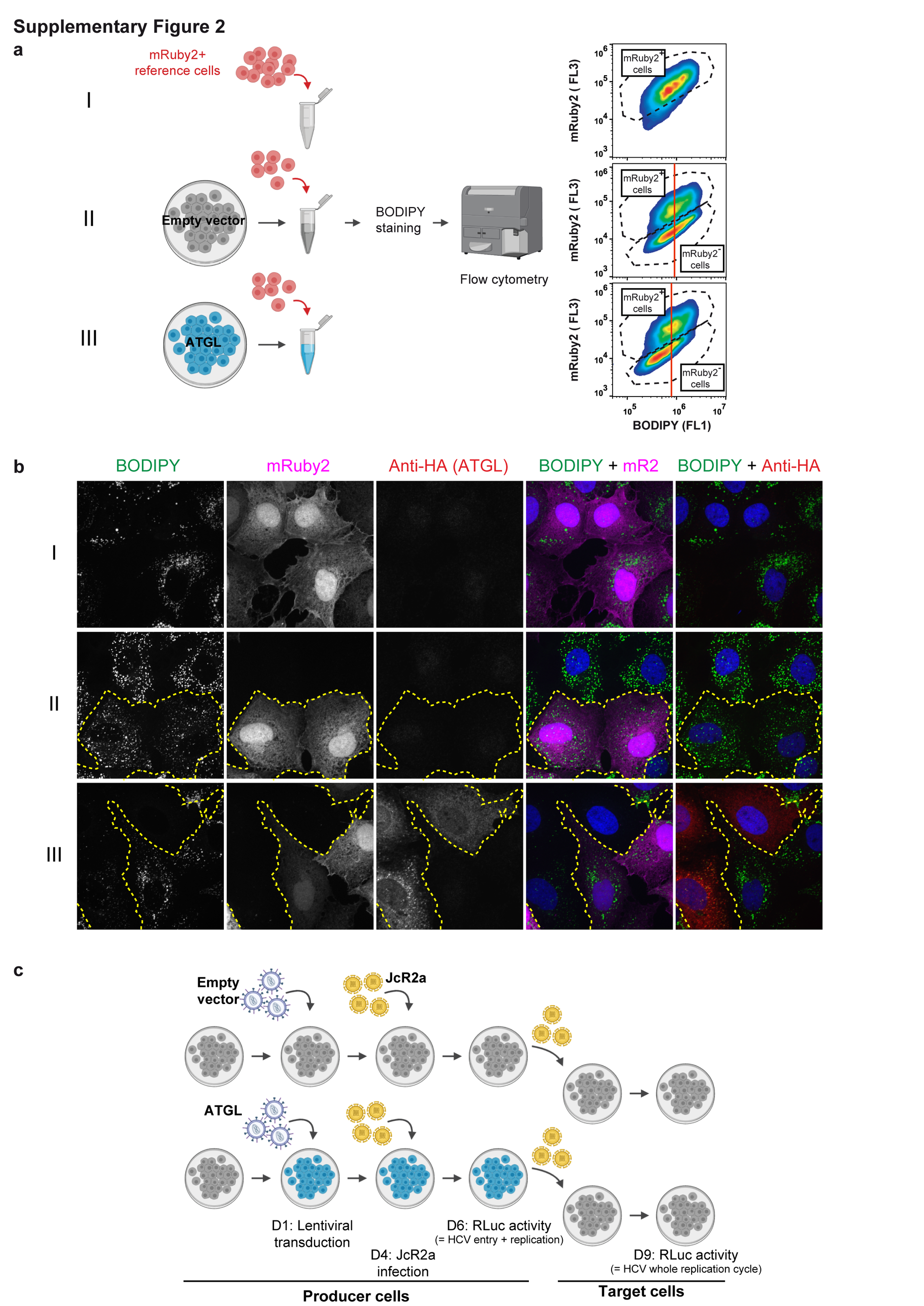

Supplement: S2 Fig — (a) Flow-cytometry-based lipid droplet lipolysis assay: principle and representative flow cytometry plots. We harvested the cells transduced with the different expression constructs (e.g. empty vector (II) or ATGL expression vector (III)) and spiked in a reference cell population that constitutively expresses mRuby2. As a quality control, we also analysed the reference cell population alone (I). We then stained the cell mixtures with the BODIPY lipid droplet dye. The cells of interest and the reference cells can be distinguished in the red channel (FL3, mRuby2, see the two cell population clouds on the 2nd and 3rd plots) and we normalized the BODIPY signal of the cells of interest to the signal of the reference cells. Representative flow cytometry plots are depicted on the right side. The vertical red line highlights the shift of the ATGL-over-expressing cell population towards the left as compared to the reference cell population, indicating a decrease in lipid droplet content (3rd plot). The cell line transduced with an empty vector on the contrary has a similar lipid droplet content as the reference cell line (2nd plot). (b) Representative microscopy pictures illustrating the strategy used in (a). The cells were transduced and mixed as in (a) but the cell mixtures were seeded on coverslips 2 days post-transduction and fixed for immunofluorescence one day later (corresponding to harvest time of the cells for flow cytometry in panel a). We stained the samples with BODIPY and Dapi and further detected the HA-tagged ATGL (detected with the anti-HA antibody and a secondary anti-mouse antibody conjugated to A647) to illustrate the ATGL expression in the mRuby2-negative cell populations. We outlined the mRuby2-positive cell population manually with a yellow dotted line. The roman numerals refer to panel a. The contrasts for the Dapi, BODIPY, and mRuby2 channels were automatically enhanced; for the HA channel (which was negative for images I and II), the intensity for al [file ppat.1008554.s002.tif]

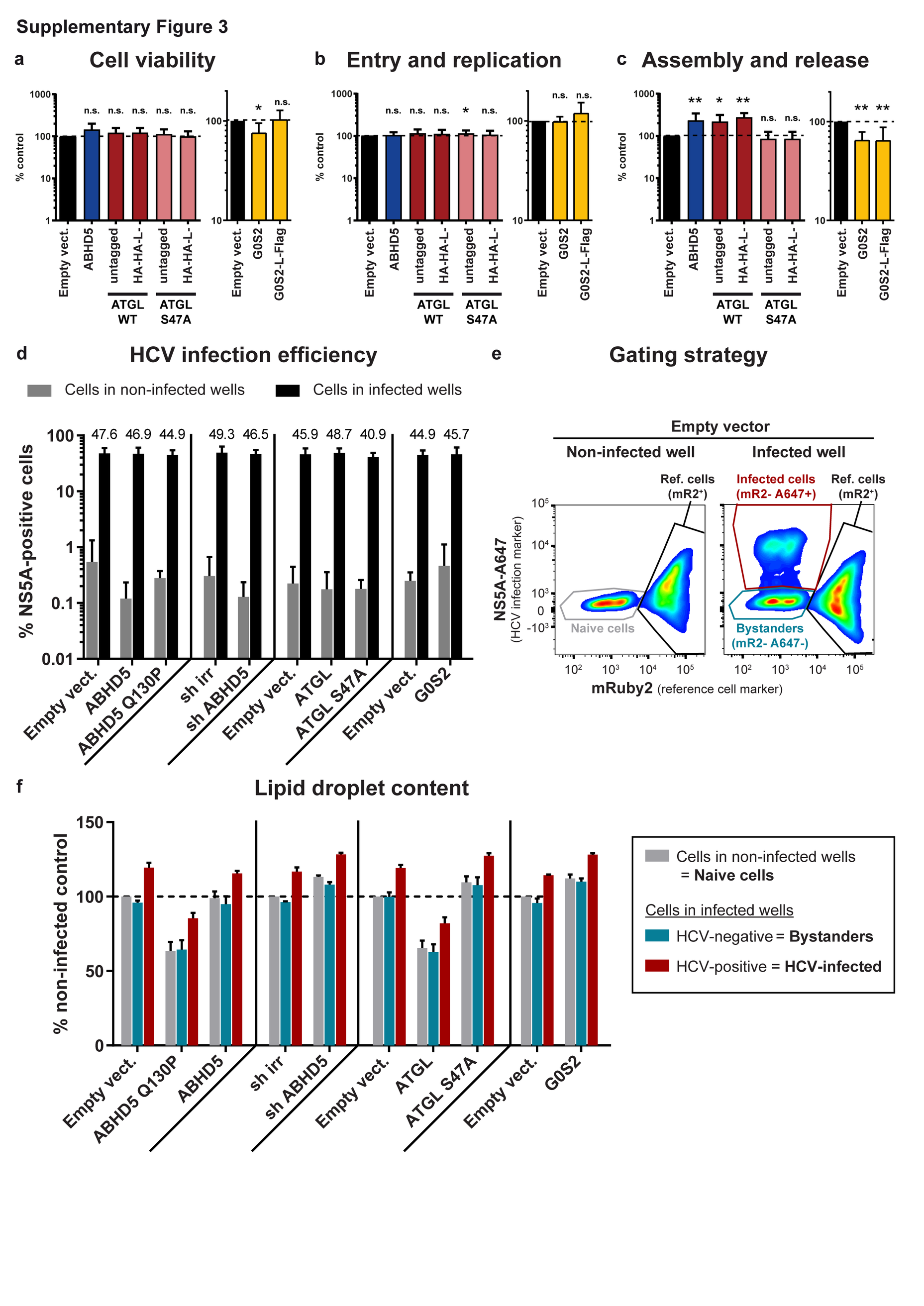

Supplement: S3 Fig — (a, b, c) Cell viability (a), HCV entry and replication (b), and HCV assembly and release (c) were assessed upon over-expression of ABHD5, ATGL or G0S2 (see Fig 4B). (a) Cell viability was assessed by measuring the Firefly luciferase (FLuc) activity in the producer cells. (b) HCV entry and replication were determined by measuring the RLuc activity in the producer cells and normalizing for any effect on cell viability (FLuc producer cells). (c) HCV assembly and release were assessed by measuring the RLuc activity in the target cells and normalizing for any effect on earlier steps of the replication cycle (RLuc in the producer cells). Note that panel c shows the same data as Fig 4B, but with a logarithmic scale, for consistency within the figure. (d) Monitoring of HCV infection rate, in the set of experiments analysed in Fig 4E and S3C Fig (n = 3). About half the cell population was successfully infected as shown by positive NS5A staining at the end of the experiment. (e) Gating strategy. Example flow cytometry plots illustrate the different cell populations and gates, with representative data from empty vector-transduced cells. In the left plot, the wells were kept non-infected. In the right plot, the cells were infected with HCV. In both cases, the cells were harvested at the end of the experiment and mixed with mRuby2-expressing reference cells, gated in black. HCV-infected cells are gated in red, the bystander cells in blue and the naive cells in grey, consistently with the colour code in panel c and in Fig 4E. (f) Effect of HCV infection status on the lipid droplet content of cells with altered ATGL activity (n = 3). The plot summarizes the same data as depicted in Fig 4E. Similarly as in Fig 4E, the lipid droplet content of the different cell populations was measured and normalized for the reference mRuby2-expressing cell population, to correct for staining or measurement variations. In this graph however, for each condition on the X axis, the data was normalize [file ppat.1008554.s003.tif]

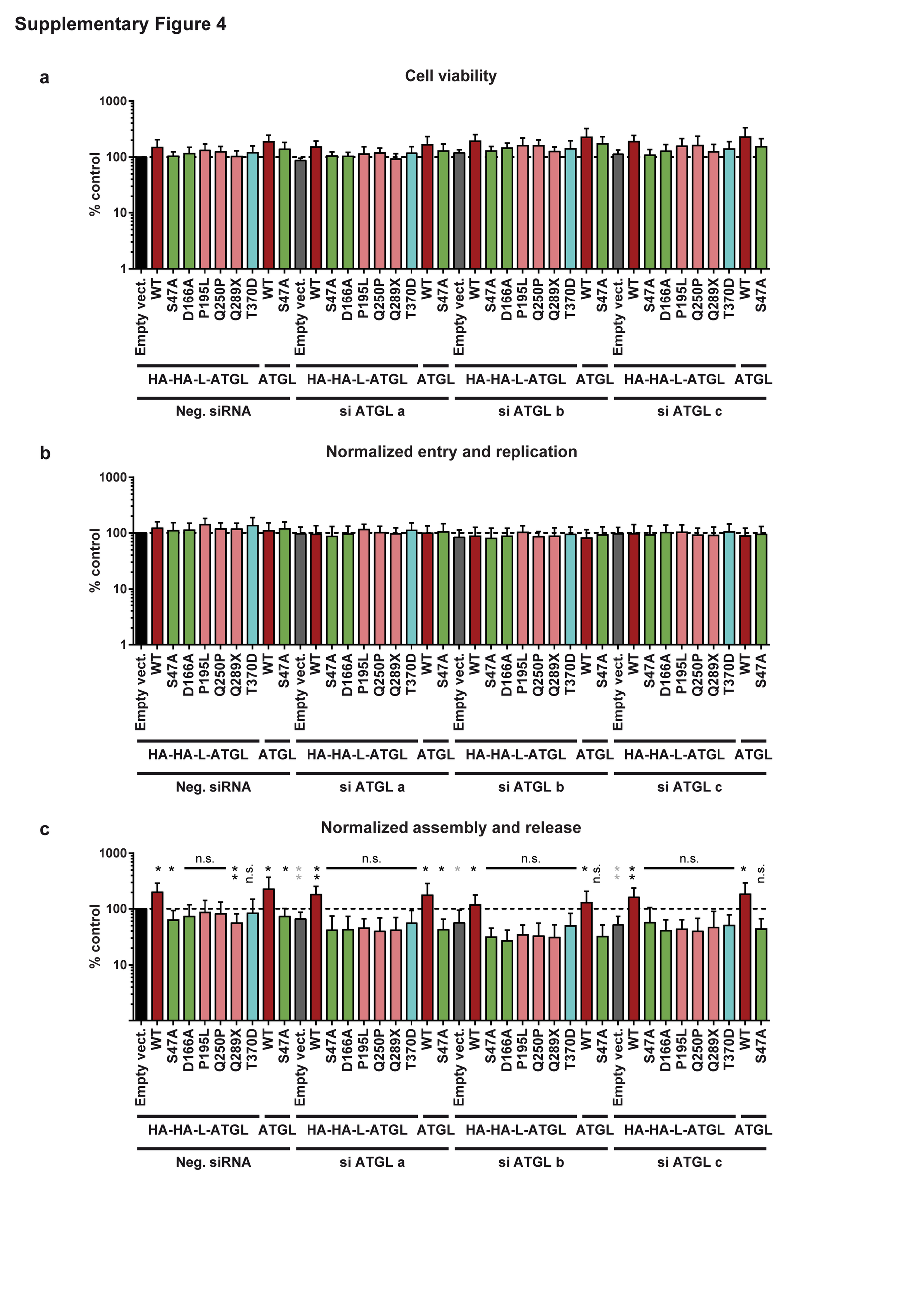

Supplement: S4 Fig — (a, b, c) Cell viability (a), HCV entry and replication (b), and HCV assembly and release (c) were assessed as described in S3A–S3C Fig. Note that panel c shows the same data as Fig 7D, but with a logarithmic scale, for consistency within the figure (n = 8). (TIF) [file ppat.1008554.s004.tif]

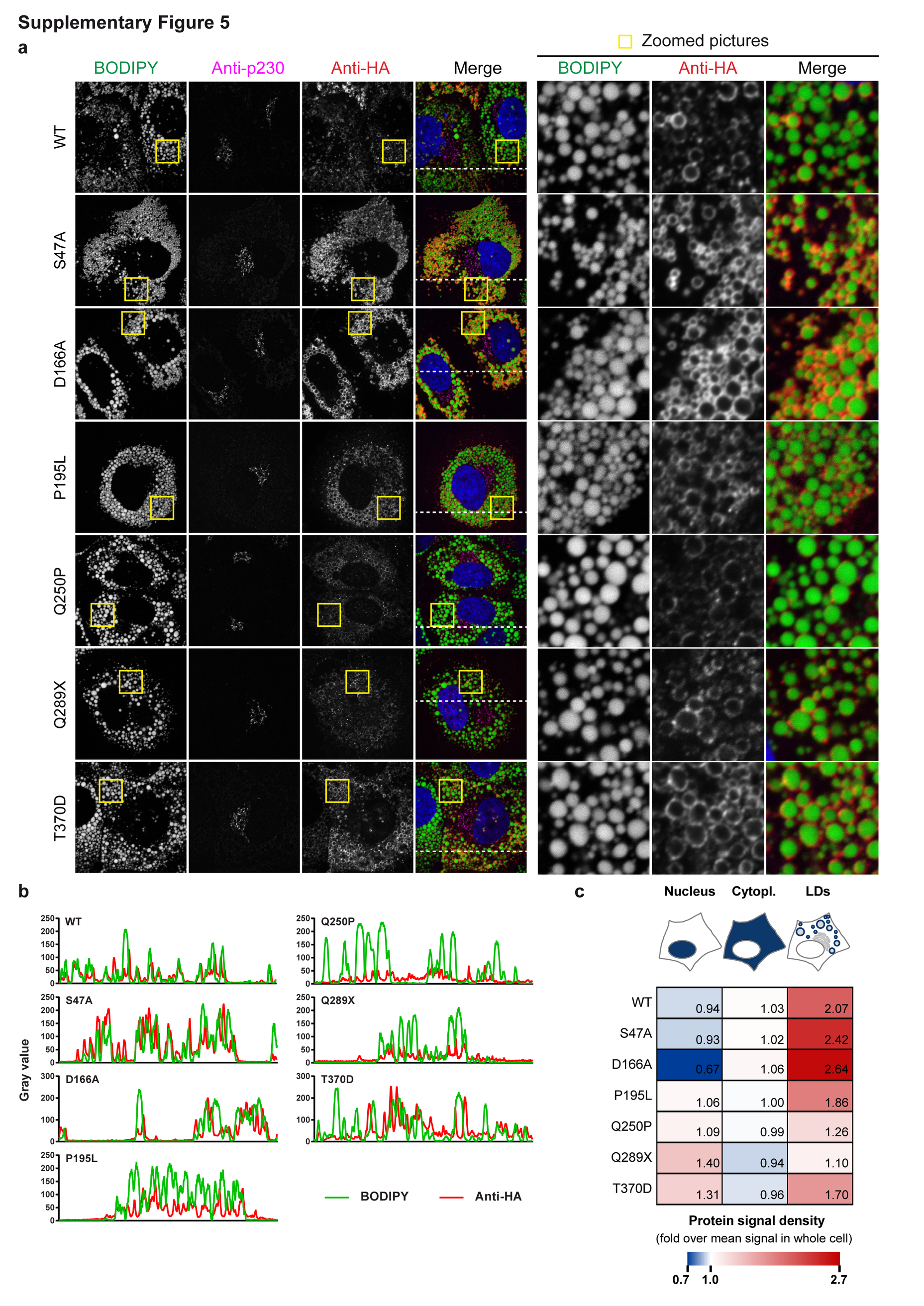

Supplement: S5 Fig — Idem Fig 8, but with oleic acid treatment of the cells to induce lipid droplet accumulation. (TIF) [file ppat.1008554.s005.tif]

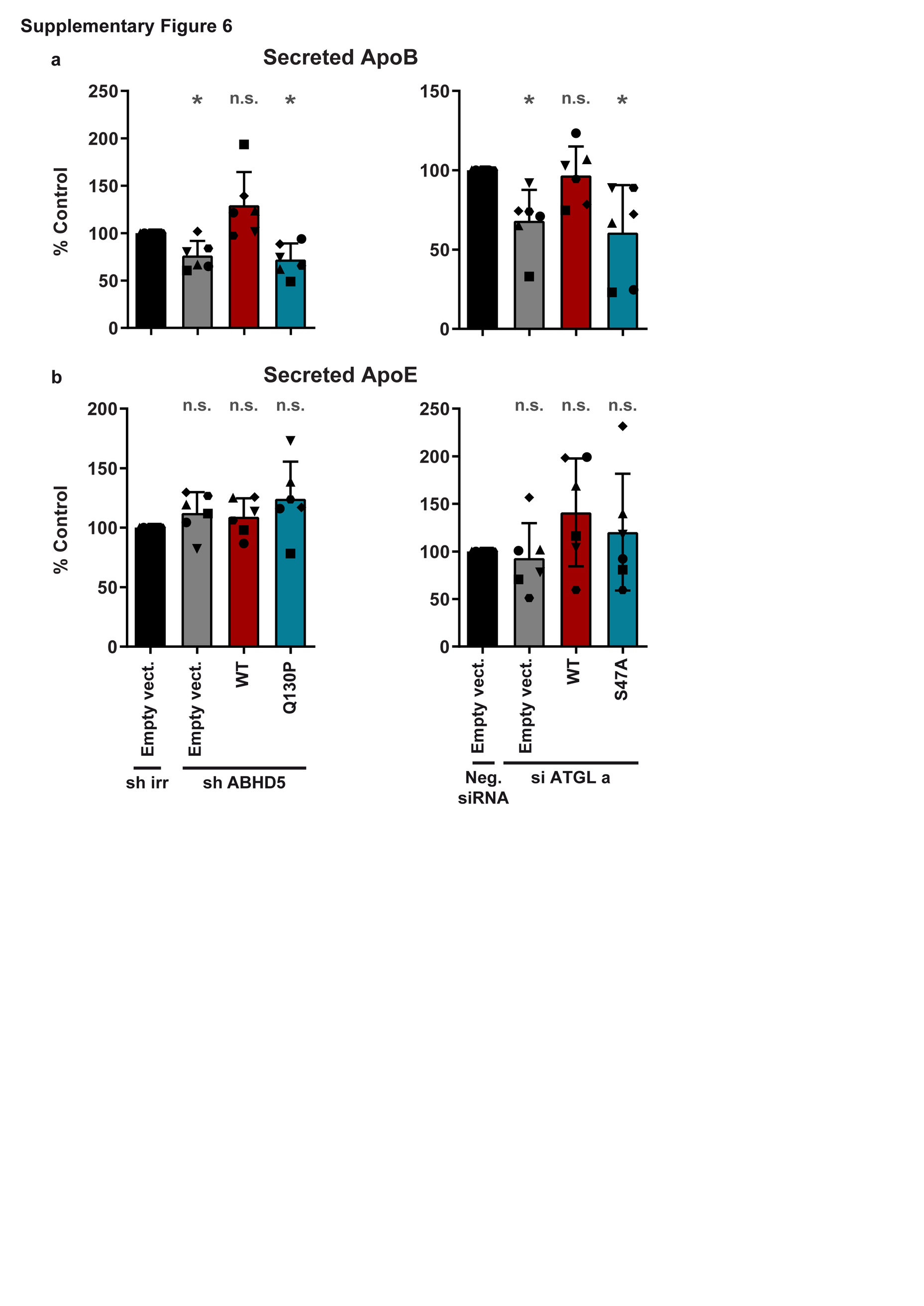

Supplement: S6 Fig — ApoB (a) and ApoE (b) levels were measured by ELISA in the supernatants of cells with manipulated ABHD5 (left half of the figure) or ATGL (right half of the figure) protein expression (n = 6). Asterisks indicate significant changes as compared to the control (first bar of each graph, in black). (TIF) [file ppat.1008554.s006.tif]

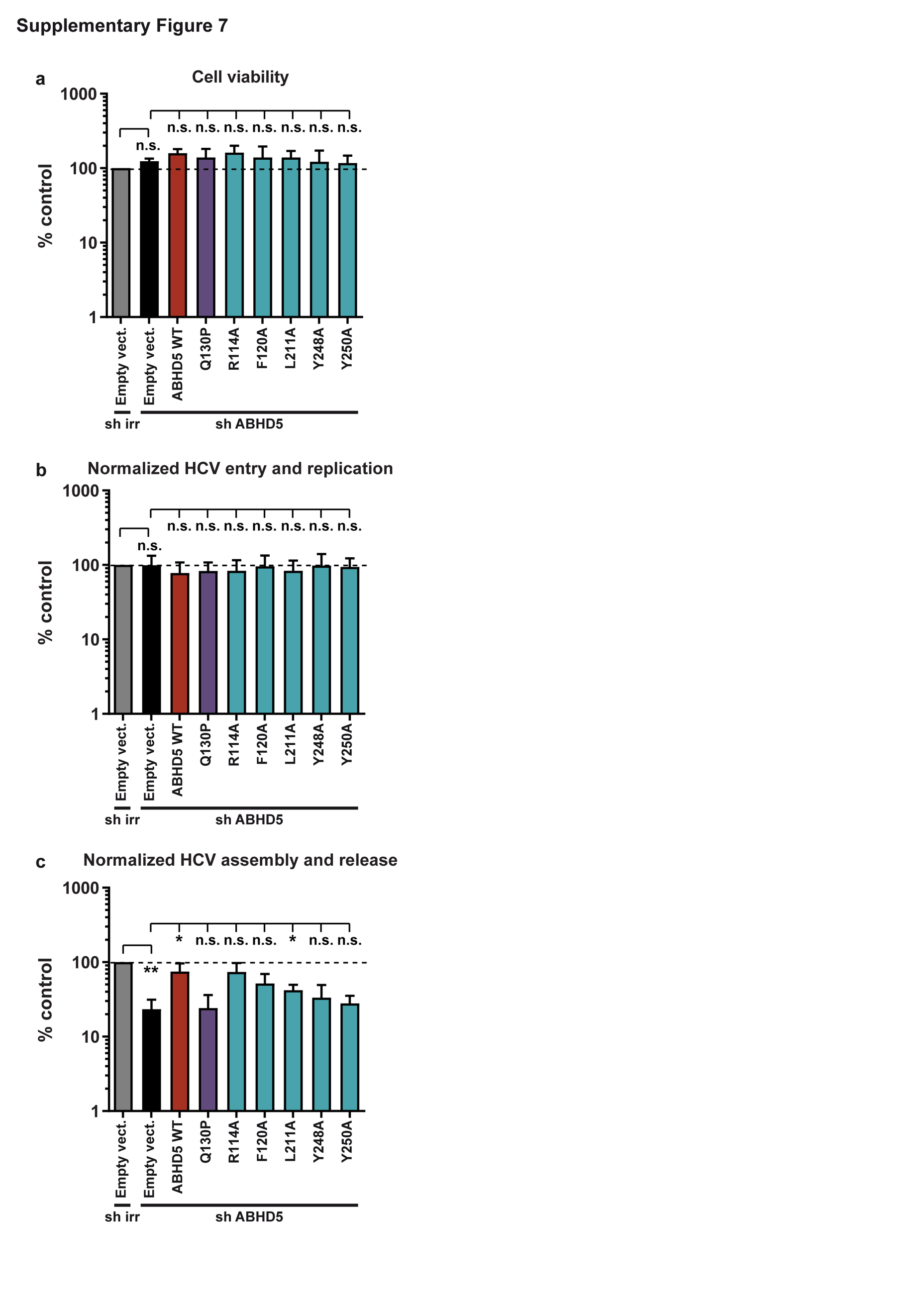

Supplement: S7 Fig — Cell viability (a), HCV entry and replication (b), and HCV assembly and release (c) were assessed as described in S3A–S3C Fig (n = 3). Note that panel c shows the same data as Fig 9D, but with a logarithmic scale, for consistency within the figure. (TIF) [file ppat.1008554.s007.tif]

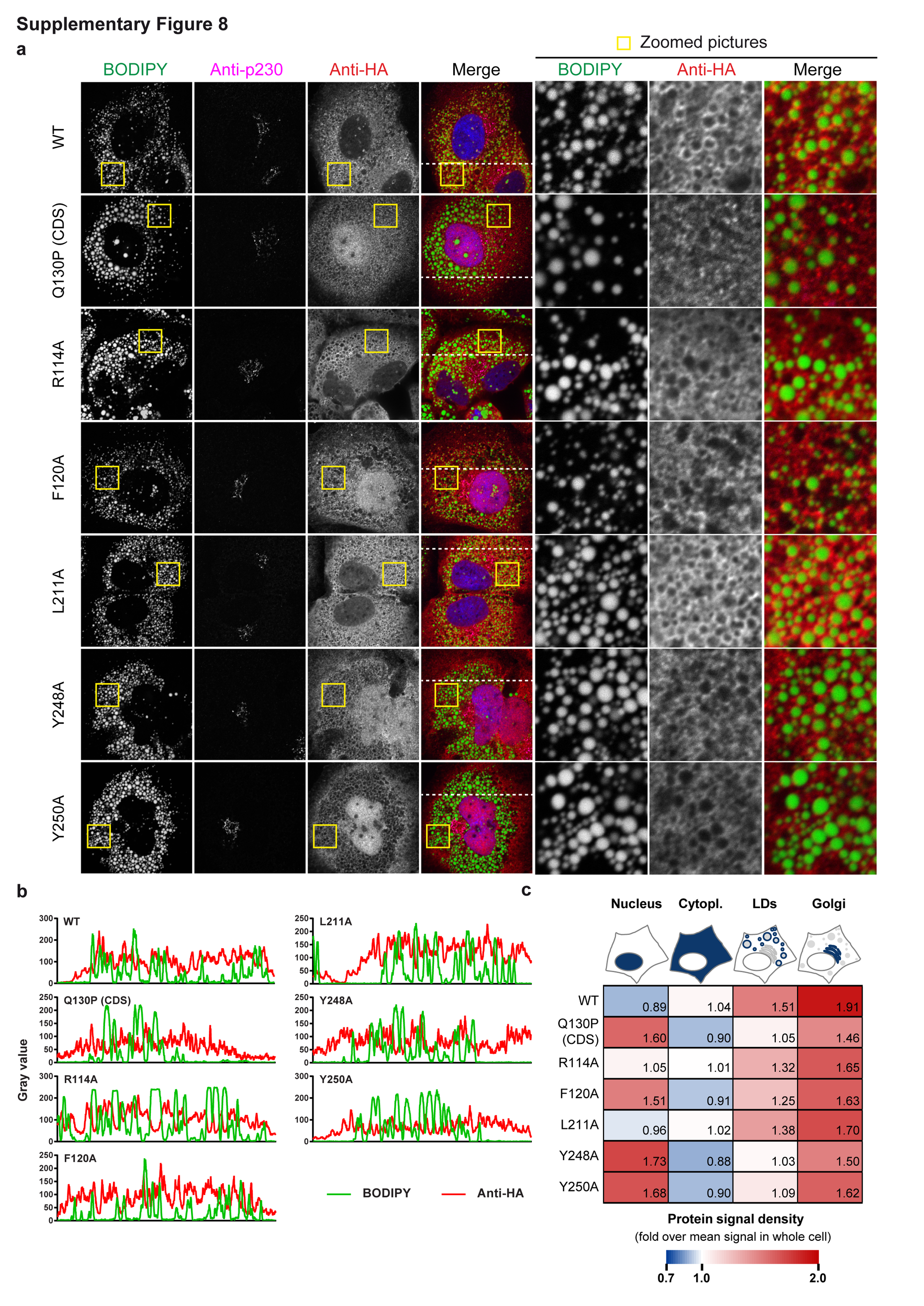

Supplement: S8 Fig — Idem Fig 10, but with oleic acid treatment of the cells to induce lipid droplet accumulation. (TIF) [file ppat.1008554.s008.tif]

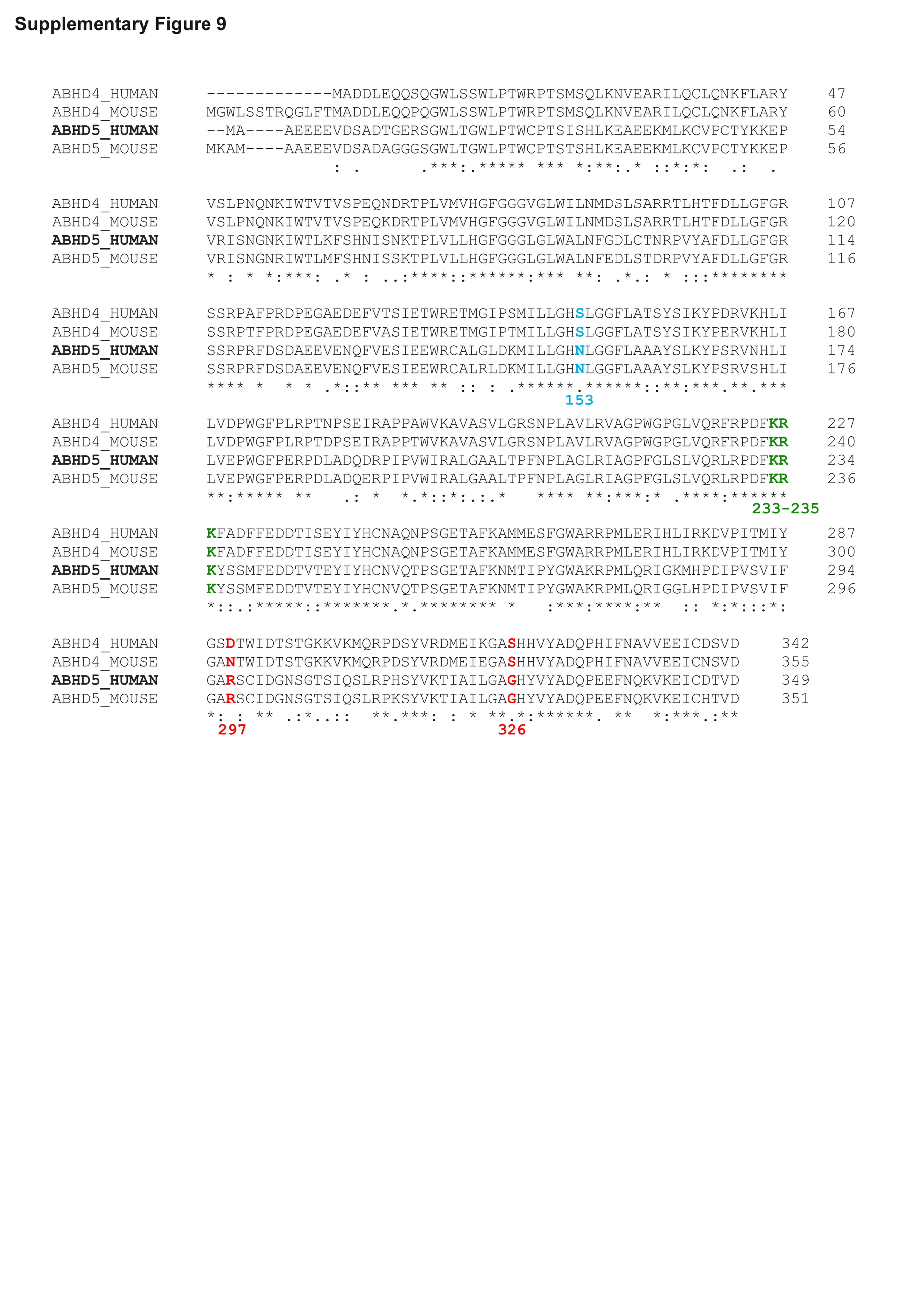

Supplement: S9 Fig — Protein sequences were aligned using the Clustal Omega tool [90] of the EMBL-EBI website (https://www.ebi.ac.uk/Tools/msa/clustalo/). The sequences correspond to the UniProt accession numbers Q8TB40 (human ABHD4), Q3U7M5 (mouse ABHD4), Q8WTS1 (human ABHD5) and Q9DBL9 (mouse ABHD5). Key residues are highlighted in colour, with their position relative to the human ABHD5 sequence indicated below. The two residues differing between ABHD4 and ABHD5 and conferring the ATGL co-factor activity [47] are indicated in red. The position of the catalytic serine residue (occupied by an asparagine residue in ABHD5) is shown in blue. The TBLC motif, conserved between ABHD4 and 5, and crucial for ABHD5 co-lipase activity [30] is in green. (TIF) [file ppat.1008554.s009.tif]

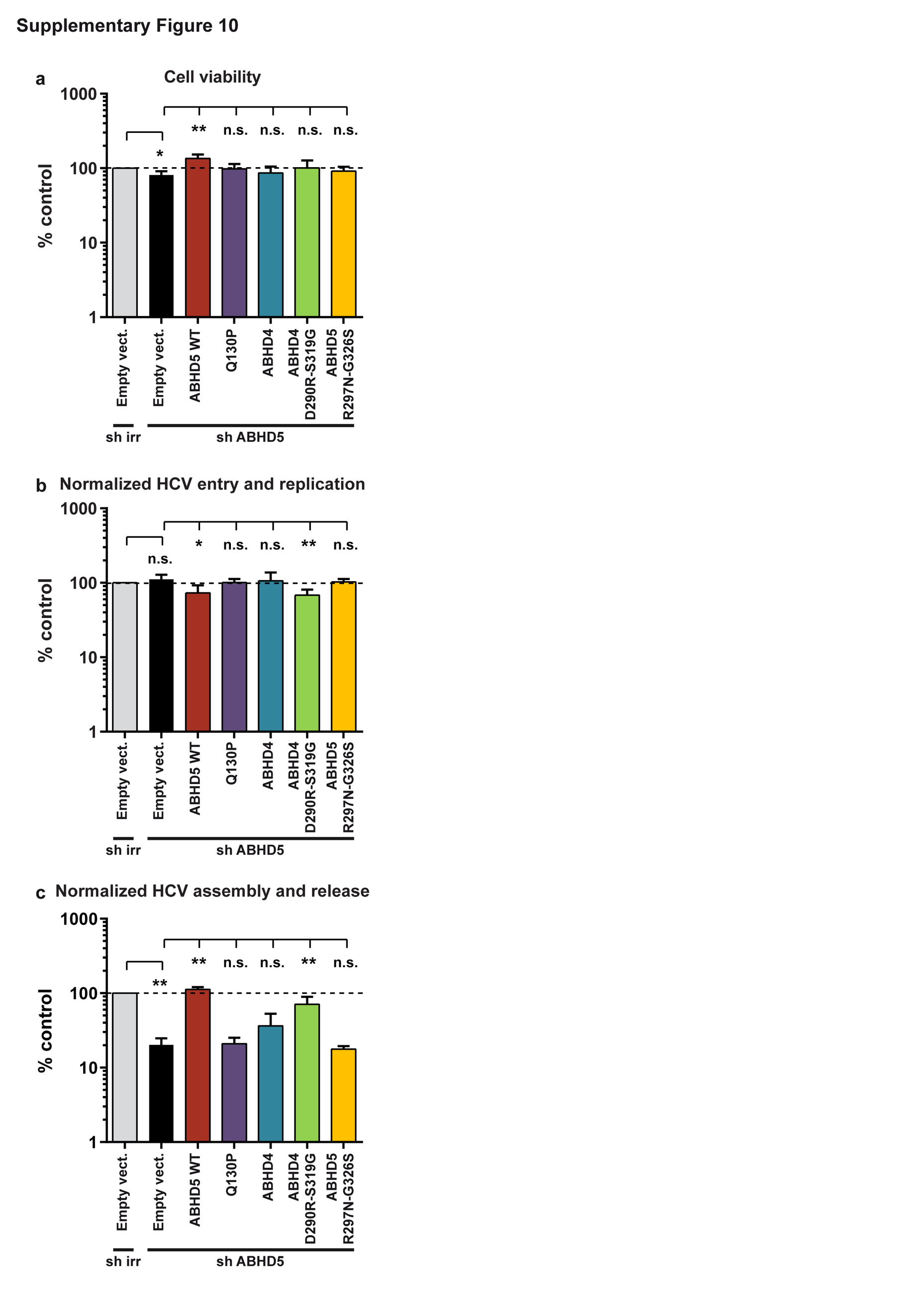

Supplement: S10 Fig — Cell viability (a), HCV entry and replication (b), and HCV assembly and release (c) were assessed as described in S3A–S3C Fig (n = 4). Note that panel c shows the same data as Fig 11D, but with a logarithmic scale, for consistency within the figure. (TIF) [file ppat.1008554.s010.tif]

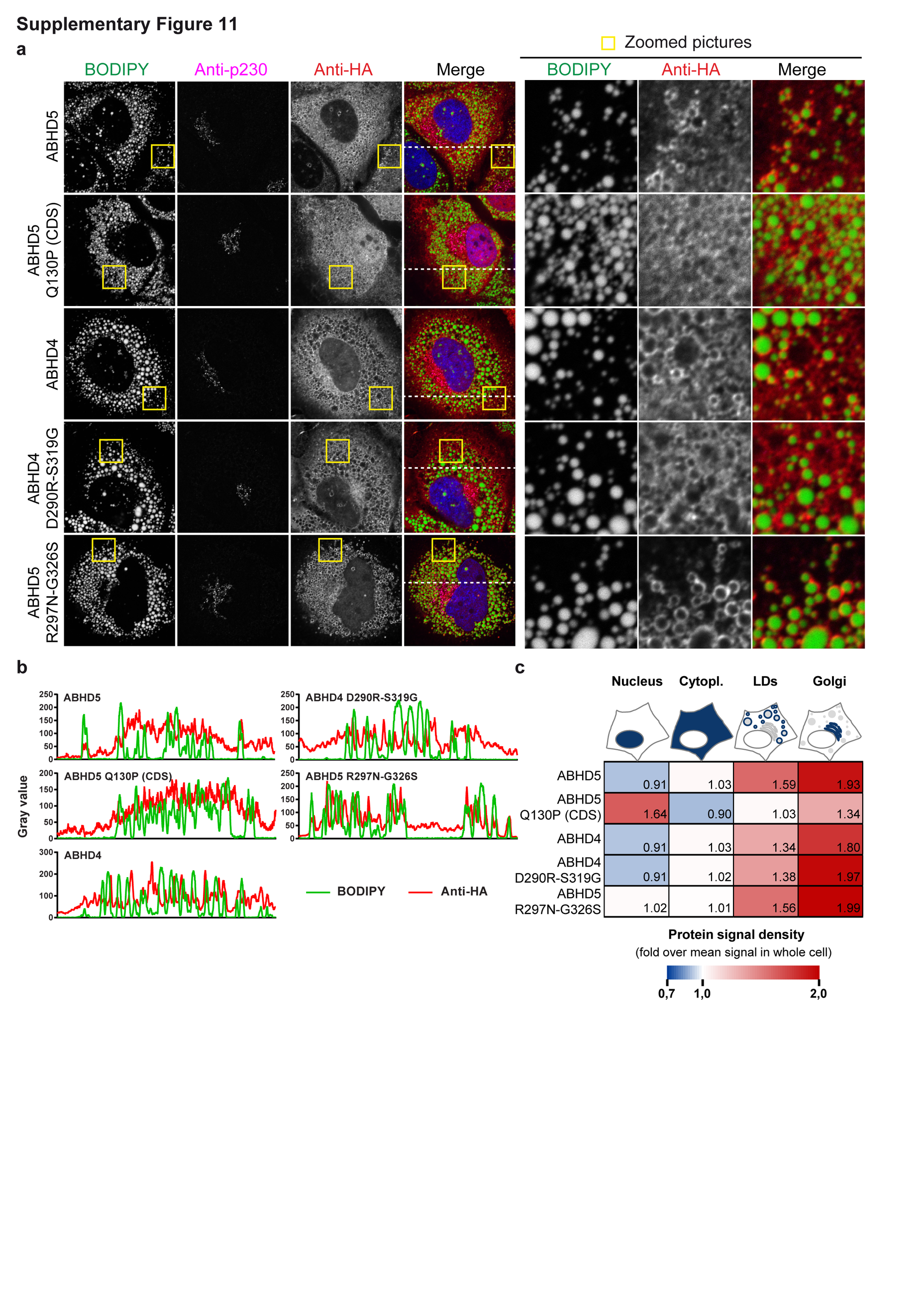

Supplement: S11 Fig — Idem Fig 12, but with oleic acid treatment of the cells to induce lipid droplet accumulation. (TIF) [file ppat.1008554.s011.tif]
